# The Interaction Between Dietary Fat Level, n-3 LC-PUFA, and Zinc on Their Postprandial Absorption Kinetics in Atlantic Salmon (*Salmo salar*)

**DOI:** 10.1155/anu/6173690

**Published:** 2024-12-28

**Authors:** HaoHang Fang, Nini H. Sissener, Øystein Sæle, Trygve Sigholt, Antony J. Prabhu Philip

**Affiliations:** ^1^Feed and Nutrition, Institute of Marine Research, Bergen, Norway; ^2^Department of Biological Sciences (BIO), University of Bergen, Bergen, Norway; ^3^BioMar AS, Havnegata 9, Trondheim 7010, Norway; ^4^Norwegian Institute of Food, Fisheries and Aquaculture Research (Nofima), Bergen, Norway

**Keywords:** *Salmo salar*, fat level, n-3 LC-PUFA, postprandial absorption kinetics, zinc

## Abstract

Two short-term feeding trials were conducted on *Salmo salar*, with the interaction between dietary zinc (Zn) and fat level in trial 1 and with the interaction between dietary Zn and n-3 long-chain polyunsaturated fatty acids (n-3 LC-PUFA) in trial 2, focusing on postprandial plasma parameters, intestinal Zn and fat uptake and transport. After 4-week feeding interventions, samples were collected at different postprandial time points, ranging from 0 to 36/38 h after feeding. Results showed that increased Zn level in feed significantly increased the postprandial plasma Zn level in trial 1 (8–9°C). On the contrary, the postprandial plasma Zn level was not affected by the dietary Zn under higher temperature conditions (trial 2, 10−12°C). Further, analyzed markers related to intestinal Zn uptake and transport were not affected by dietary fat level and n-3 LC-PUFA. In addition, analyzed markers related to intestinal fat uptake and transport were not affected by dietary Zn. Intestinal Zn transport plays a key role in regulating body Zn storage, while intestinal fat transport influences lipid accumulation within the intestine. Understanding how these processes respond to dietary components is critical for maintaining fish health and welfare.

## 1. Introduction

Atlantic salmon (*Salmo salar*) is the most important aquaculture species in Norway. In order to reduce feed costs and develop sustainable feed, high-energy diets and the replacement of marine-based ingredients with plant-based ingredients have been widely applied in Norwegian salmon feed in recent years [1]. These strategies have resulted in an increase in the fat level, a decrease in the n-3 long-chain polyunsaturated fatty acids (n-3 LC-PUFA) level, and decreases in the zinc (Zn) level and availability in feed [1, 2].

Zn is crucial for multiple physiological functions in fish, such as forming Zn-containing enzymes and promoting skeletal development [3, 4]. Feeding fish with Zn-deficient diets induced Zn-deficiency diseases, such as lens cataracts, skin erosion, and skeletal malformation [5–7]. Considering reductions in Zn level and availability in plant-based feeds, additional Zn must be provided to meet the Zn requirement in *S. salar*. However, Zn digestibility is lower than 40%, and Zn retention is less than 30% in *S. salar* [2], indicating that a high level of Zn in the feed would have a negative environmental impact. In this regard, the EU limited the upper Zn level in salmon feed to 180 mg kg^−1^, and the EFSA has suggested further reducing it to 150 mg Zn kg^−1^ [8, 9]. Therefore, it is necessary to increase knowledge on enhancing Zn availability and utilization in salmon feeds.

Even though fish can osmotically absorb aqueous minerals, feed remains the predominant resource to maintain body minerals homeostasis [10]. In the case of Zn, the absorptive site is primarily the intestine [10]. Similar to mammals, fish have two families of cellular Zn transporters: zips (Zrt/Irt-like proteins, transport Zn into cytosol) and znts (Zn transporters, transport Zn out of the cell) [11]. In addition, because zip4 and znt1 are located at the apical and basolateral epithelium, respectively, they are regarded as two key proteins in intestinal Zn uptake and transport [11]. After Zn enters the cell, metallothionein (mt) would bind with Zn to prevent Zn toxicity [12]. If cellular Zn levels exceed the mt binding capacity, transcription factors such as metal regulatory transcription factor 1 (mtf1) would be upregulated, thus increasing the expression of znt1 and promoting the Zn efflux [12]. However, very little is known about the regulation of Zn transporters in Atlantic salmon.

Intestinal Zn uptake and transport are affected by several factors, including anti-nutrient factors (such as phytate), mineral interactions (with iron [Fe] and manganese [Mn]), chemical form of Zn (chelated vs. inorganic) [10, 13]. Furthermore, dietary component interaction might be another factor impacting intestinal Zn uptake and transport, such as fat level and n-3 LC-PUFA. In mammals, higher plasma and femur Zn levels were observed in rats fed with 3% fat diets compared to those in the 26% fat diet group [14]. Also, rats fed with fish oil-based diet (rich in n-3 PUFA) obtained higher hepatic Zn levels than those fed with a lard-based diet (rich in saturated fatty acids [SFA]), indicating that n-3 PUFA may have a positive effect on Zn absorption [15]. A recent study in Atlantic salmon also reported that fish fed with low-fat/high n-3 LC-PUFA diets obtained the higher whole-body Zn level compared to those fed with high-fat/low n-3 LC-PUFA diets [16]. Due to the crucial physiological role and the upper legal limitations in feed, understanding the Zn uptake as affected by dietary components is important to the aquaculture industry.

Furthermore, dietary Zn may impact intestinal fat transport. According to a previous study, rats fed Zn-deficient diets obtained lower triacylglycerol (TAG) absorption and abundant intestinal lipid droplets, demonstrating the importance of Zn on intestinal fat transport [17, 18]. However, the effect of dietary Zn on intestinal fat transport in fish is still unclear.

In previous fish nutrition studies, samples were generally collected at fasting status or 24 h after the final meal. Worth mentioning, different postprandial sampling time points may obtain different plasma minerals [19] and TAG [20] levels, indicating the importance of the sampling time selected when investigating plasma parameters. However, reports on postprandial plasma profiles related to lipids and minerals in *S. salar* are still scarce. Therefore, the aim of this study was to investigate the combined effect of dietary fat level and Zn, and the combined effect of dietary n-3 LC-PUFA and Zn on postprandial plasma profiles in *S. salar*. In addition, intestinal gene expression related to Zn and lipid uptake and transport in selected postprandial time points were also analyzed.

## 2. Materials and Methods

### 2.1. Experimental Diets

Diet formulations and analyzed nutritional compositions are shown in Table 1. In trial 1, four experimental diets containing one of two different levels of fat (%) and Zn (mg kg^−1^) were formulated and referred to as follows (fat/Zn): HFHZ, 35/200; HFLZ, 35/120; LFHZ, 32/200; LFLZ, 32/120. In comparison, Norwegian *S. salar* commercial feeds for growing out contain 35%–39% fat level [21]. Additionally, the EU restricts the maximum Zn in *S. salar* feed to 180 mg kg^−1^ [9], whereas 120 mg Zn kg^−1^ is a suboptimal level in *S. salar* plant-based feed [22]. Fat levels in the feed were adjusted by varying the levels of fish oil and rapeseed oil, with wheat gluten, guar meal, and wheat meal used to balance the composition. Zn levels in the diet were modified by adding Zn sulfate at different doses.

In trial 2, three experimental diets were used. The first two experimental diets (HFHZ and HFLZ) were the same as trial 1, while the third diet contained higher n-3 LC-PUFA (with high fat and Zn levels). Three diets were named as follows (eicosapentaenoic acid [EPA] + docosahexaenoic acid [DHA], % of total feed/Zn, mg kg^−1^): HFHZ, 2.0/200; HFLZ, 2.0/120; HPUHZ, 3.2/200 (average level of DHA + EPA in Norwegian commercial salmon feed is 2.2% (2020) [1]. Different dietary n-3 LC-PUFA levels between HFHZ and HPUHZ were achieved by adjusting the ratio of rapeseed oil and fish oil. In addition, due to the lack of cholesterol (CHO) in rapeseed oil, additional CHO was provided in HFHZ, HFLZ, LFHZ, and LFLZ to obtain a similar CHO level as the HPUHZ diet.

### 2.2. Fish Feeding and Management

Two feeding trials were performed at the Matre Research Station of the Institute of Marine Research in Norway. All the sampling procedures were performed on euthanized fish. The study was evaluated by the animal experimentation administration of IMR (Forsksdyrforvaltningen) and approved as a noninvasive animal study conducted in accordance with Norwegian regulations on the use of animals in research in line with the EU directive 2010/63/EU. This trial was exempt from an animal ethics approval (FOTS application) to the Norwegian Food Safety Authority, according to the regulation “FOR-2015-06-18-761 Regulation concerning the use of animals for scientific purposes, § 6. Godkjenning av forsøk.”

**Figure 1:**
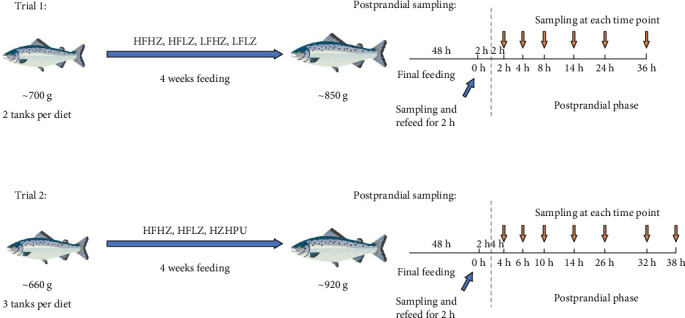
Schematic diagram of feeding management and postprandial sampling in this manuscript.

In both feeding trials, *S. salar* were fed a commercial feed (Skretting Norway) for 2 weeks to acclimate them to experimental conditions. In trial 1 (Figure 1), 280 *S. salar* with IW of 718 ± 58 g were randomly distributed to eight tanks (1350 L, dimensions 1.5 × 1.5 × 0.8 m), with 35 fish in each. Four experimental diets (HFHZ, HFLZ, LFHZ, and LFLZ) were randomly assigned to the tanks in duplicates. Fish were provided experimental diets twice per day until apparent satiation (9:00 and 16:00) for 4 weeks. During the nutritional intervention, the fish were held in full-strength seawater with the water temperature ranged from 8 to 9°C.

In trial 2 (Figure 1), 225 fish with a weight of 667 ± 20 g were randomly distributed to nine tanks (1350 L, dimensions 1.5 × 1.5 × 0.8 m) with 25 fish in each. Three experimental diets (HFHZ, HFLZ, and HPUHZ) were randomly assigned to nine tanks in triplicates. Further, fish were fed with one of three experimental diets twice daily until apparent satiety (9:00 and 16:00) for 4 weeks. During the nutritional intervention, the fish were held in full-strength seawater, and the water temperature ranged from 10 to 12°C.

### 2.3. Postprandial Sampling

As shown in Figure 1, at the termination of the feeding trial, fish were fasted for 48 h to completely empty their gastrointestinal tract, which was considered their preprandial status (0 h), and their blood and intestine were sampled. Next, fish were fed with respective diets for 2 h, and the postprandial time was calculated after this feeding. Similar samples (blood and mid-intestine) were collected at different postprandial time points. In trial 1, five fish were sampled from each tank (total 10 fish from each dietary treatment group) at 2, 4, 8, 14, 24, and 36 h after feeding. In trial 2, three fish from each tank (total nine fish from each dietary treatment group) were sampled at 4, 6, 10, 14, 26, 32, and 38 h after the feeding.

During the sampling, fish were randomly and carefully removed from the tank to reduce stress and then euthanized with an overdose of tricaine mesylate (MS-222, Tricaine Pharmaq). The euthanized fish were measured for weight and length, following which blood was drawn from the caudal vein using a lithium heparin-coated vacutainer. The blood samples were centrifuged (14,200 g, 2 min, 4°C) to obtain supernatant (plasma), which was then frozen on dry ice for plasma parameters analysis. Afterward, the mid intestine (just behind the pyloric ceca) was collected after emptying the intestinal contents and immediately frozen in liquid nitrogen for further analysis. During the sampling, the state of feed remnants in the stomach was inspected and recorded, which served as the indicator of fish ingestion. The number of fish without pellets in their stomachs is presented in Table S1. If the fish hadn't eaten, the sample was excluded from further analysis.

### 2.4. Chemical Composition Analysis

The chemical composition in the feed was analyzed after homogenization. Moisture, protein, and ash were analyzed by drying to constant weight (105°C), the Kjeldahl method (N x 6.25), and muffle furnace (550°C), respectively; fat level in feeds was analyzed by acid hydrolysis and extraction with diethyl ether.

FAs composition in feed was analyzed as described in [23]. Briefly, after isolation of lipids from feed (Folch solution) and completely dried chloroform–methanol phase (using N_2_ gas), residual lipids were trans-methylated overnight with 2′-2′-dimethoxy propane, methanolic HCl, and benzene at room temperature. Methyl esters were isolated and analyzed using a gas chromatograph (Hewlett Packard 6890; HP) equipped with a split injector, an SGE BPX70 capillary column (SGE Analytical Science), and a flame ionization detector. He_2_ served as the carrier gas, with the injector and detector temperatures both maintained at 280°C. The oven temperature was initially increased from 50 to 180°C at a rate of 10°C per minute, then further increased to 240°C at a rate of 0.7°C per minute. Individual FA methyl esters were identified by comparison with previously characterized standards. Results were further analyzed using HP ChemStation software. FAs results were presented as a percentage of the total fatty acids in the feed, and the absolute amount of EPA + DHA per gram of feed was calculated using C23:0 methyl ester as the internal standard.

Mineral contents (Zn, copper [Cu], Fe, Mn, and selenium [Se]) in diet and plasma were measured using inductively coupled plasma mass spectrometry (ICP-MS), as described in [24]. Specifically, samples (0.2 g dry feed with 0.5 mL deionized water or 0.5 mL plasma) and 2 mL concentrated HNO_3_ were added into test tubes and digested in a Milestone UltraWave Microwave Digestion System (Milestone Inc., USA). Further, digested samples were diluted to 25 mL by deionized water. Next, minerals were analyzed in ICP-MS (Thermo Scientific, USA) equipped with an autosampler (FAST SC-4Q DX, Elemental Scientific, USA). The eluate was introduced into the nebulizer tube of the ICP-MS, and minerals were analyzed in the KED reaction mode. To correct for instrumental drift during the analysis, a solution of germanium and rhodium was added online. The ICP-MS was tuned prior to analysis using a 1 ppb tuning solution B (Thermo Fisher, in 2% HNO3 and 0.5% HCl). Data collection and processing were carried out using Qtegra software (Thermo Scientific). An external calibration curve ranging from 10 to 500 ng mL^−1^ was utilized to quantify the minerals.

Plasma lipids, including TAG, CHO, high-density lipoprotein (HDL), low-density lipoprotein (LDL), and total protein (TP), were measured using Pentra C400 (HORIBA; Montpellier, France), following the instruction of the manufacturer.

### 2.5. Gene Expression

Intestinal samples for gene expression analysis were selected at three postprandial time points based on the plasma TAG profile: exogenous lipid had not entered circular system (trial 12 h; trial 2:4 h), exogenous lipid entered circular system (trial 1 and trial 2:14 h) and exogenous lipid abundantly entered circular system (trial 1:24 h; trial 2:26 h).

Candidate genes for qPCR included two house-keeping genes (*β*-actin and elongation factor 1 alpha [ef-1*α*]), five genes related to Zn transport (solute carrier family 39 member 4 [zip4], metallothionein A [mta], metallothionein B [mtb], solute carrier family 30 member 1 [znt1], mtf1) and 11 genes related to lipid transport and metabolism (Niemann-Pick C1-like 1 [npc1l1], cluster of differentiation 36 [cd36], fatty acid transporter protein 4 [fatp4], fatty acid transporter protein 6 [fatp6], fatty acid-binding protein 2 [fabp2], diglyceride acyltransferase 1 [dgat1], microsomal triglyceride transfer protein [mtp], apolipoprotein B [apob], carnitine O-palmitoyltransferase 1 [cpt1], monoacylglycerol O-acyltransferase 2 [mgat2], apolipoprotein A-IV [apoa4]).

Total RNA from intestine samples was isolated using the Maxwell HT simplyRNA Kit (Promega, USA) and the Biomek 4000 automated liquid handler (Beckman Coulter, USA), following the instructions of the manufacturer. In addition, the quality and quantity of isolated RNA were measured in the Bioanalyzer (Agilent 2100) and the spectrophotometer (NanoDrop ND-1000), respectively. Further, an inverse transcription kit (Thermo Fisher Scientific, USA) was used to synthesize cDNA, following the instructions of the manufacturer. Afterward, RT-PCR for target genes was quantified on the qPCR instrument (Bio-Rad, USA) with SYBR GREEN PCR Master Mix (Roche-Norge, Norway) [25], with the following program: 10 min preincubation at 95°C, followed by 40 cycles of 95°C for 30 s, 60°C for 30 s, 72°C for 30 s, and a melting curve. The expression level of the target gene was normalized on the CFX Maestro (Bio-Rad, USA) based on two house-keeping genes. The associated information of candidate genes is listed in Table 2. Except for *β*-actin and ef-1*α* [26], other primers were newly designed through NCBI online primer designing tool (https://www.ncbi.nlm.nih.gov/tools/primer-blast/index.cgi?). One-step RT-PCR (QIAGEN one-step RT-PCR kit) was used to evaluate the primer specificity, according to the instructions of the manufacturer.

### 2.6. Statistical Analysis

The normality and variance of the data homogeneity were analyzed under the Shapiro–Wilk test and Levene's test (*p*  > 0.05), respectively. In trial 1, the weight gain (WG) was analyzed by two-way ANOVA, with fat level and Zn as independent variables (*n* = 2). In addition, results from other growth performance parameters (initial weight [IW], initial condition factor [ICF], final weight [FW], final condition factor [FCF]) were analyzed by “two-way nested ANOVA,” with fat level and Zn as independent variables (tank as a random factor). Furthermore, results from plasma minerals, plasma lipids, and intestinal gene expression were analyzed by three-way nested ANOVA, with fat level, Zn, and time as independent variables (tank as a random factor), and the significant results between “0h” and “selected time point” were analyzed by multiple comparisons with “Dunnett's test” (5 samples from each tank, total 10 samples from each dietary treatment). In trial 2, the WG was analyzed by *t*-test between the HFHZ and HFLZ (or HFHZ vs. HPUHZ) groups (*n* = 3). In addition, results from other growth performance parameters (IW, ICF, FW, FCF) were analyzed by *t*-test nested between the HFHZ and HFLZ (or HFHZ vs. HPUHZ) groups (tank as a random factor). Furthermore, results from plasma minerals, plasma lipids, and intestinal gene expression were analyzed by two-way nested ANOVA, with diet (Zn or n-3 LC-PUFA) and time as independent variables (tank as a random factor), and the significant results between “0h” and “selected time point” were also analyzed by multiple comparisons with “Dunnett's test” (three samples from each tank, total nine samples from each dietary treatment). If the *p*-value was <0.05, results between groups were regarded as significant. All experimental results were presented as the mean ± standard deviation (SD). Statistical analysis was performed in the software GraphPad Prism 8 (Insightful Science, USA) and R (R Development Core Team, 2011), respectively.

## 3. Result

### 3.1. Growth Performance

The growth performance of *S. salar* fed the different diets from trial 1 and trial 2 are presented in Table 3. In trial 1, the WG of the fish ranged from 18.5% to 22.2%, while in trial 2, it ranged from 39.6% to 44.9%. In addition, the survival rates (SRs) of fish fed with different diets were 100% in trial 1 and 94%–98% in trial 2. However, 4-week dietary treatments did not impact the fish growth among all experimental groups in trial 1 and trial 2 (*p*  > 0.05).

### 3.2. Postprandial Plasma Minerals

**Figure 2:**
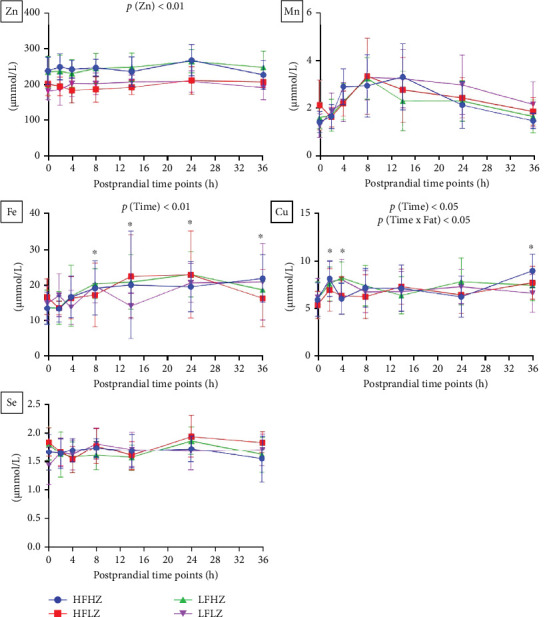
Postprandial plasma mineral profiles of *Salmo salar* as affected by dietary fat level and Zn after 4-weeks feeding (trial 1); each point in the figure represents the mean ± SD (*n* = 10); *⁣*^*∗*^ significant results compared to preprandial results (0h). Cu, copper; Fe, iron; Mn, manganese; Se, selenium; Zn, zinc.

In trial 1, reduced Zn level in feed significantly reduced the postprandial plasma Zn level (*p*  < 0.01, Figure 2, all results presented in Table S2). Additionally, postprandial plasma Fe (*p*  < 0.01) and Cu (*p*  < 0.05) profiles were significantly impacted by the time change. Peak values of plasma Fe (14–24 h, *p*  < 0.01) and Cu (2−4 h and 36 h, *p*  < 0.05) appeared at specific postprandial time points. Further, the postprandial plasma Cu profile was significantly affected by Time x Fat (*p*  < 0.05). Compared to low-fat diet treatment, high-fat diet treatments reduced the plasma Cu level at postprandial 4 h but increased the plasma Cu level at postprandial 36 h. On the contrary, postprandial plasma profiles of Zn, Mn, Fe, and Se were not affected by dietary fat level (*p*  > 0.05).

**Figure 3:**
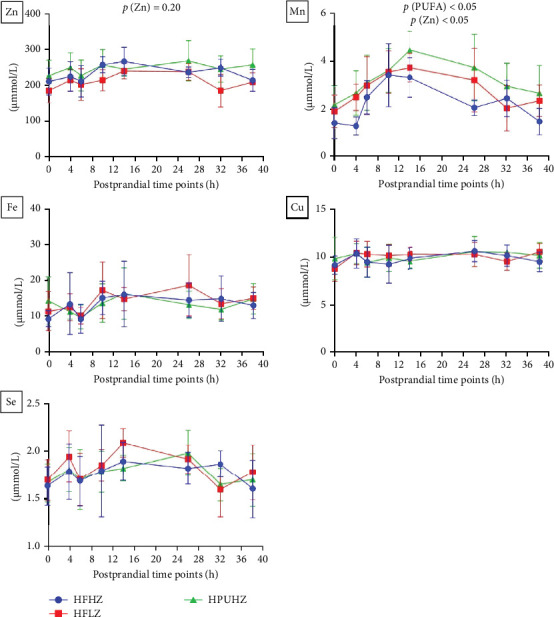
Postprandial plasma mineral profiles of *Salmo salar* as affected by dietary Zn and n-3 LC-PUFA after 4-weeks feeding (trial 2); each point in the figure represents the mean ± SD (*n* = 9). Cu, copper; Fe, iron; Mn, manganese; n-3 LC-PUFA, n-3 long-chain polyunsaturated fatty acids; SD, standard deviation; Se, selenium; Zn, zinc.

In trial 2, the postprandial plasma Zn profile was not affected by dietary Zn (*p*=0.20) (Figure 3, all results presented in Table S3). In addition, HFHZ treatment significantly reduced the postprandial plasma Mn level compared to HFLZ treatment (*p*  < 0.05). In addition, fish fed with the high n-3 LC-PUFA diet obtained significantly higher postprandial plasma Mn level compared to those fed with the low n-3 LC-PUFA diet (*p*  < 0.05), which is attributed to different Mn levels in diets (Table 1). However, postprandial plasma profiles of Zn, Fe, Cu, and Se were not affected by dietary n-3 LC-PUFA (*p*  > 0.05).

### 3.3. Postprandial Plasma Lipids

**Figure 4:**
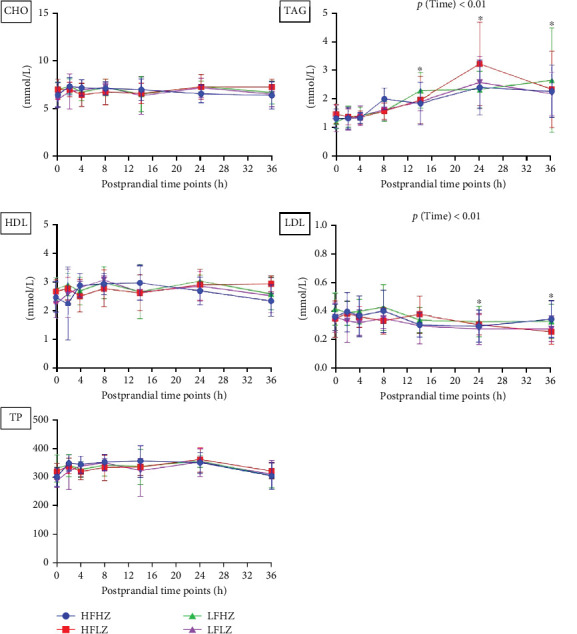
Postprandial plasma lipid profiles of *Salmo salar* as affected by dietary fat level and Zn after 4-weeks feeding (trial 1); each point in the figure represents the mean ± SD (*n* = 10); *⁣*^*∗*^ significant results compared to preprandial results (0h). CHO, cholesterol; HDL, high-density lipoprotein; LDL, low-density lipoprotein; SD, standard deviation; TAG, triglyceride; TP, total protein; Zn, zinc.

The postprandial plasma TAG profile was significantly impacted by the time changes in trial 1 (*p*  < 0.01, Figure 4, all results presented in Table S2). After 14 h of feeding, the plasma TAG level had risen significantly (*p*  < 0.01) compared to that at preprandial (0 h). Further, the peak value of plasma TAG appeared at postprandial 24 h and was statistically elevated to 36 h (*p*  < 0.01). However, dietary fat level and Zn did not impact the postprandial plasma TAG profile (*p*  > 0.05).

**Figure 5:**
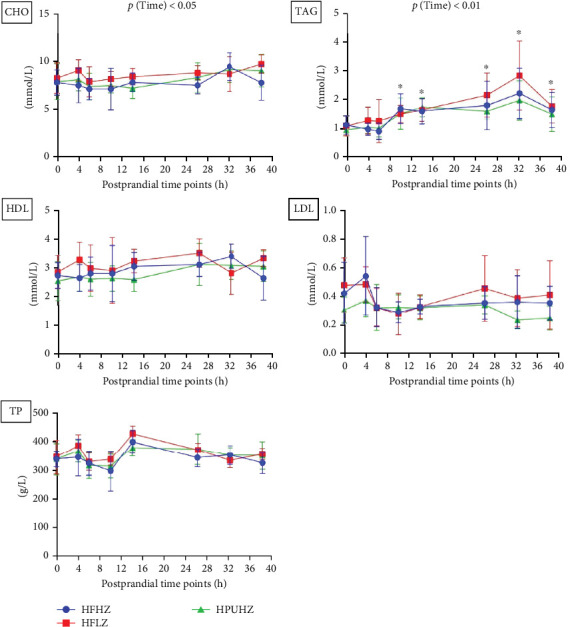
Postprandial plasma lipid profiles of *Salmo salar* as affected by dietary Zn and n-3 LC-PUFA after 4-weeks feeding (trial 2); each point in the figure represents the mean ± SD (*n* = 9); *⁣*^*∗*^ significant results compared to preprandial results (0 h). CHO, cholesterol; HDL, high-density lipoprotein; LDL, low-density lipoprotein; n-3 LC-PUFA, n-3 long-chain polyunsaturated fatty acids; SD, standard deviation; TAG, triglyceride; TP, total protein; Zn, zinc.

In trial 2, the postprandial plasma TAG profile was significantly affected by the time change (*p*  < 0.01, Figure 5, all results presented in Table S3), which agrees with the result in trial 1. Compared to preprandial status (0 h), plasma TAG level was significantly higher between 10 and 38 h after the meal (*p*  < 0.05). Also, plasma CHO was affected by postprandial time (*p*  < 0.05), and the result at postprandial 32 h was significantly higher than that at postprandial 6 h. However, the postprandial plasma TAG profile was not affected by dietary n-3 LC-PUFA and Zn (*p*  > 0.05).

### 3.4. Postprandial Intestinal Gene Expression Related to Zn Uptake and Transport

**Figure 6:**
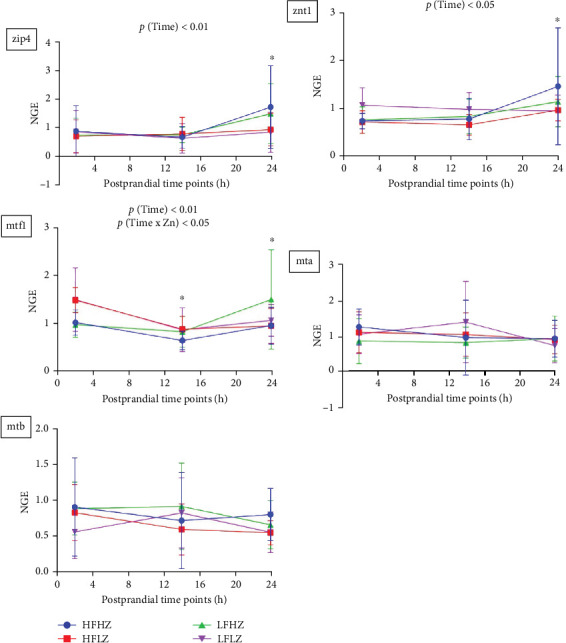
Postprandial intestinal mRNA expression related to Zn transport in *Salmo salar* as affected by dietary fat level and Zn (trial 1); intestinal Zn uptake: zip4; intracellular Zn transportation: mta, mtb; Zn efflux: znt1, mtf1; values mean ± SD (*n* = 10). *⁣*^*∗*^ Significant results compared to results at 2 h. NGE: normalized gene expression; SD, standard deviation; Zn, zinc.

As shown in Figure 6 (trial 1, all results presented in Table S4), intestinal mRNA expression related to Zn uptake and transport was not affected by dietary Zn and fat level (*p*  > 0.05). However, gene expression related to Zn uptake and transport, including *zip4* (*p*  < 0.01), *znt1* (*p*  < 0.05), and *mtf1* (*p*  < 0.01), were significantly affected by postprandial timing. Compared to the 2 h postprandial mark, *zip4*, *znt1*, and *mtf1* were upregulated at 24 h.

**Figure 7:**
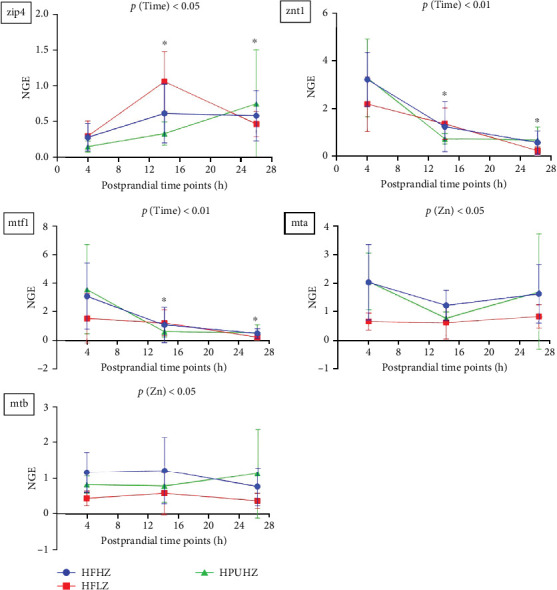
Postprandial intestinal mRNA expression related to Zn uptake and transport in *Salmo salar* as affected by dietary Zn and n-3 LC-PUFA (trial 2); intestinal Zn uptake: zip4; intracellular Zn transportation: mta, mtb; Zn efflux: znt1, mtf1; values mean ± SD (*n* = 9). *⁣*^*∗*^Significant results compared to results at 4 h. n-3 LC-PUFA, n-3 long-chain polyunsaturated fatty acids; NGE: normalized gene expression; SD, standard deviation; Zn, zinc.

In trial 2, reduced Zn level in feed significantly downregulated the intestinal mRNA expression of *mta* and *mtb* postprandially (*p*  < 0.05, Figure 7, all results presented in Table S5). In addition, postprandial timing significantly impacted intestinal mRNA expression related to Zn uptake and transport, including *zip4* (*p*  < 0.05), *znt1* (*p*  < 0.01), and *mtf1* (*p*  < 0.01). At 26 h postprandially, *zip4* was upregulated, while *znt1* and *mtf1* were downregulated compared to the 4 h mark.

### 3.5. Postprandial Intestinal Gene Expression Related to Lipid Uptake and Transport

**Figure 8:**
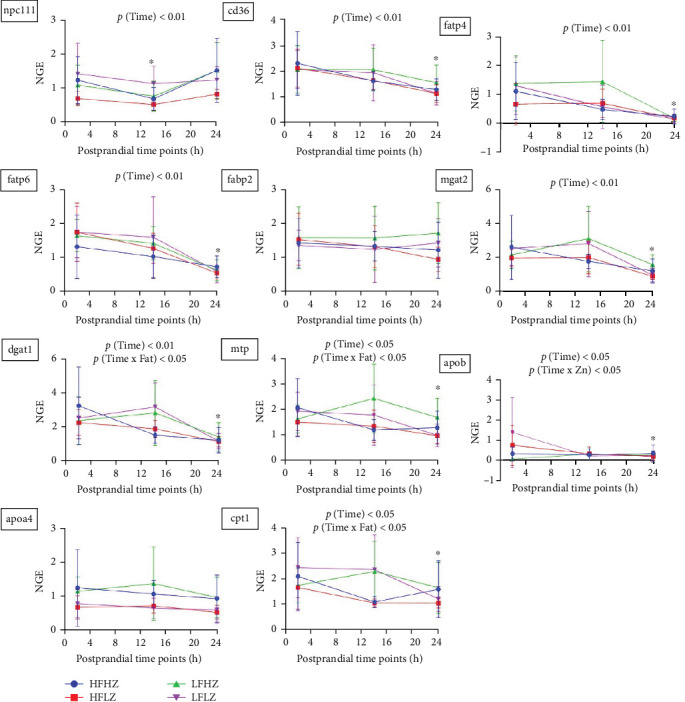
Postprandial intestinal mRNA expression related to lipid uptake and transport in *Salmo salar* as affected by dietary fat level and Zn (trial 1); intestinal FAs uptake: cd36; intestinal CHO uptake: npc1l1; intracellular FA transportation: fatp4, fatp6, fabp2; TAG re-esterification: mgat2, dgat1; lipoprotein formation: mtp, apob, apoa4; FA *β*-oxidation: cpt1; values are mean ± SD (*n* = 10). *⁣*^*∗*^ Significant results compared to results at 2 h. CHO, cholesterol; FAs, fatty acids; NGE, normalized gene expression; SD, standard deviation; TAG, triglyceride.

In trial 1, intestinal mRNA expression related to lipid uptake and transport was not significantly affected by dietary Zn (*p*  > 0.05, Figure 8, all results presented in Table S4). However, the expression levels of *dgat1*, *mtp*, and *cpt1* were significantly influenced by the interaction between time and fat levels (*p*  < 0.05), with reduced fat level downregulating the expression of these genes at 14 h postprandially. Additionally, most mRNA expressions related to lipid uptake and transport were significantly impacted by postprandial timing (*p*  < 0.05). Compared to postprandial 2 h, CHO uptake gene (*npc1l1*) was downregulated at 14 h, and genes related to TAG uptake and transport (*cd36*, *fatp4*, *fatp6*, *mgat2*, *dgat1*, *mtp*, *apob*) and fatty acid *β*-oxidation (*cpt1*) were downregulated at 24 h.

**Figure 9:**
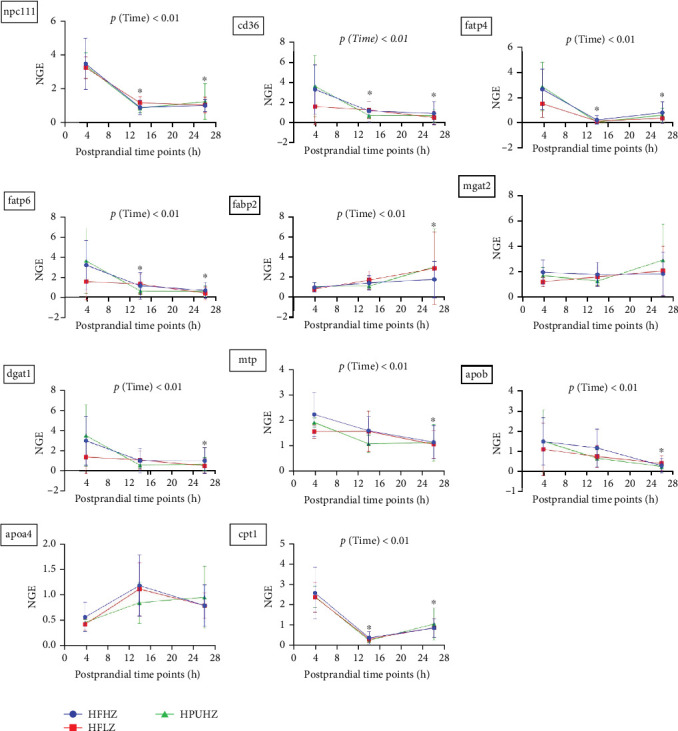
Postprandial intestinal mRNA expression related to lipid uptake and transport in *Salmo salar* as affected by dietary n-3 LC-PUFA and Zn (trial 2); intestinal FAs uptake: cd36; intestinal CHO uptake: npc1l1; intracellular FA transportation: fatp4, fatp6, fabp2; TAG re-esterification: mgat2, dgat1; lipoprotein formation: mtp, apob, apoa4; FA *β*-oxidation: cpt1; Values are mean ± SD (*n* = 9). *⁣*^*∗*^ Significant results compared to results at 4 h. CHO, cholesterol; FA, fatty acids; n-3 LC-PUFA, n-3 long-chain polyunsaturated fatty acids; NGE, normalized gene expression; SD, standard deviation; TAG, triglyceride; Zn, zinc.

In trial 2, dietary Zn did not impact intestinal mRNA expression related to lipid uptake and transport (*p*  > 0.05, Figure 9, all results presented in Table S5), consistent with the results observed in trial 1. However, postprandial timing had a significant impact on most of these gene expressions (*p*  < 0.01). At 26 h postprandially, genes related to lipid transport and metabolism, including *npc1l1*, *cd36*, *fatp4*, *fatp6*, *fabp2*, *dgat1*, *mtp*, *apob*, and *cpt1*, were downregulated compared to the 4 hr mark.

## 4. Discussion

### 4.1. Postprandial Kinetics of Zn and Other Minerals

Increasing knowledge on the postprandial absorption kinetics of Zn and its dietary impact could improve the understanding on dietary Zn availability. Different from reports in rainbow trout [19, 27] and hybrid striped bass [28], there did not appear a significant absorptive peak in the postprandial plasma Zn profile in the present two trials. In the above-mentioned studies, one or more of the diets contained dietary Zn levels that were deficient or very low, resulting in very low basal plasma Zn concentration and hence inducing a visible peak in plasma Zn immediately after a meal. In trial 1, although postprandial plasma Zn concentration was correlated with dietary Zn level, as reported in other fish at fasting status [29–31], mRNA expression related to intestinal Zn uptake (*zip 4*) and transport (*znt 1*) was not affected by dietary Zn. These results are due to the passive uptake mechanism in the intestine. It is well known that active uptake mechanisms in the intestine, primarily DMTs, ZIPs, and ZnTs, are upregulated at limiting dietary Zn concentrations, whereas at optimal or high dietary Zn levels, passive uptake mechanisms are dominating [32, 33]. Apart from intestinal Zn absorption, other Zn metabolic processes, such as storage and excretion, are also attributed to the regulation of plasma Zn status in vertebrates [34]. In contrast with trial 1 (8–9°C), the postprandial plasma Zn was not affected by dietary Zn in trial 2 (10–12°C), which is due to the different water temperatures. As reported by Bervoets, Blust, and Verheyen [35], increased temperature would increase metal diffusion and reaction rates, resulting in increased metal accumulation in tissues. Also, increased temperature and dietary Zn level interactively increased hepatic Zn accumulation in Pelteobagrus fulvidraco [36]. In trial 2, increased Zn level in feed increased the intestinal mt (*mta* and *mtb*) expression, indicating that more exogenous Zn is temporarily stored in the intestinal epithelium by binding with mt before transport into plasma [12], and thus the postprandial plasma Zn level was not affected by dietary Zn. Temperature-dependent intestinal Zn transport was also reported in other fish studies [37–39]. These results suggest the importance of the regulatory effect of the intestine on plasma Zn [40, 41]. Therefore, a mismatch between Zn absorptive markers in the intestine and plasma Zn status at the different time-points can be attributed to the differential regulative mechanisms.

Besides the differential Zn regulation of absorptive epithelia and systemic regulation, marine fish present different intestinal Zn adaptations when subjected to different dietary Zn levels [42]. In the two present trials, intestinal mRNA expression related to Zn influx (*zip 4*) and efflux (*mtf 1* and *znt 1*) were not affected by dietary Zn, indicating the dynamic balance between intestinal Zn influx and efflux. In comparison, *Sparus aurata* (marine fish) fed with Zn-deficient diets (7.9 mg Zn kg^−1^) increased intestinal Zn influx but reduced intestinal Zn efflux to maintain intestinal Zn homeostasis, whereas fish fed optimum Zn diet (64.7 mg Zn kg^−1^) maintained the dynamic balance between intestinal Zn influx and efflux [42]. These results indicate that suboptimal Zn supplementation in *S. salar* feed (120 mg Zn kg^−1^) does not impair the dynamic balance between intestinal Zn influx and efflux after 1-month feeding trial. In addition, increased n-3 LC-PUFA or reduced fat level in feed increased whole-body Zn content in *S. salar* [16]. However, intestinal Zn uptake and transport were not affected by dietary n-3 LC-PUFA and fat level in the present study. Also, the postprandial plasma Zn profile was not affected by fat level or n-3 LC-PUFA. These results suggest that dietary fat level and n-3 LC-PUFA might impact other Zn metabolism processes (such as storage and excretion) rather than intestinal absorption, resulting in impacting whole-body Zn status.

Apart from Zn, postprandial plasma levels of other divalent trace minerals were also affected in the studies. In trial 1 (8–9°C), the postprandial plasma peak values of Fe and Cu appeared later than the report in *Oncorhynchus mykiss* (17.5°C) [19], which is due to different experimental temperatures. Increased water temperature in an appropriate range is generally associated with an increased passage rate of chyme through the gastrointestinal tract of fish [19, 27, 43]. In addition, two discontinuous peak values of postprandial plasma Cu were observed in trial 1, which is similar to the report in *O. mykiss* [19]. It can be explained that the Cu absorption site in *S. salar* occurs in the stomach, mid-intestine, and posterior intestine, similar to the report in *O. mykiss* [44]. Furthermore, increased fat level in feed reduced plasma Cu level at postprandial 4 h but increased plasma Cu level at postprandial 36 h, indicating increased dietary fat level depressed Cu absorption in the stomach but promoted Cu absorption in the intestine. Increased fat level in feed significantly increased the Cu utilization in rats (2% vs. 8% fat level) [45]. However, knowledge on the effect of fat level on Cu availability in animals is still scarce. In addition, mineral interaction is another important factor impacting mineral status in fish [13]. In trial 2, postprandial plasma Mn level was negatively correlated with dietary Zn level, which may be due to the competitive effect between Zn and Mn during absorption. In the case of Mn, its active transport during absorption is mediated by the divalent metal transporter 1 (DMT1), which also transports other divalent metals, such as Zn, Fe, and Cu [46]. Also, previous studies reported that high Zn treatment significantly reduced intestinal Fe [47] and Cu [48] absorption in *O. mykiss*. In the present study, postprandial plasma Fe and Cu were not affected by dietary Zn, indicating that *S. salar* fed with diets ranging from 120 to 200 mg Zn kg ^−1^ did not impact Fe and Cu absorption.

### 4.2. Postprandial Lipids Kinetics

The pyloric ceca and mid-intestine are the two sites for lipid digestion and absorption in *S. salar* [49, 50]. In the present study, the plasma TAG began to significantly increase at postprandial 14 h (trial 1, 8−9°C) /10 h (trial 2, 10−12°C), indicating the time point when chyme started to enter the pyloric ceca and the exogenous lipid enters the circulation system [51, 52]. Although a small proportion of plasma TAG may be attributed to hepatic VLDL secretion, lipid metabolism in the liver was not analyzed in the current study. In addition, the peak value of plasma TAG appeared at postprandial 24−36 h (trial 1, 8–9°C) and 32 h (trial 2, 10 −12°C), respectively, indicating the majority of chyme had reached the pyloric ceca and mid-intestine.

FAs are taken up and transported by the intestine through membrane-associated proteins, although a small proportion is performed by passive diffusion [53, 54]. In the two present trials, intestinal mRNA expression related to lipid uptake and transport was not affected by dietary Zn. Additionally, postprandial plasma TAG level was not affected by dietary Zn. In previous studies, Zn-deficient diet treatment increased intestinal fat accumulation in rats [17, 18], and it is correlated with inhibition of intestinal lipoprotein formation [55]. Also, Zn-deficiency impaired intestinal lipid transport in *S. aurata* [42]. Therefore, these results suggest that the 120 mg Zn kg^−1^ in *S. salar* feed is not low enough to impact intestinal lipid transport (compared to the 200 mg Zn kg^−1^ diet).

In trial 1, the postprandial plasma TAG was not affected by dietary fat level, suggesting that dietary fat level (32%–35%) did not alter the intestinal TAG transport. Similarly, a recent *S. salar* study reported that increased fat level in the feed from 16% to 25% did not impact the mRNA expression of the key lipoprotein assembly gene (*mtp*) [56]. However, in trial 1, reduced fat level in feed upregulated intestinal mRNA expression related to TAG re-esterification (*dgat1*), lipoprotein assembly (*mtp*), and *β*-oxidation (*cpt1*) at postprandial 14 h, when the chyme entered the pyloric intestine. These results may be linked to the higher wheat gluten content in the low-fat diet compared to the high-fat diet (17.5% vs. 10.2%). A previous study showed that increasing dietary wheat gluten from 15% to 30% elevated the intestinal gene expression associated with lipid metabolism and transport in *S. salar* [57]. However, it is important to note that mRNA expression may not directly correspond to protein synthesis levels due to regulatory processes such as mRNA editing, modification, and degradation [58]. In trial 1, the downregulation of *dgat1*, *mtp*, and *cpt1* genes at postprandial 24 h compared to 14 h suggests possible mRNA degradation. Future studies could provide more comprehensive insights by simultaneously quantifying mRNA and protein expression.

In the two present trials, the postprandial plasma CHO level was not affected by the dietary Zn, fat level, and n-3 LC PUFA, which is attributed to the similar CHO level among different experimental diets, as reported in other fish studies [59, 60]. Similarly, changing the dietary fat level (31% vs. 38% fat level) [61] or fatty acid profiles [62] did not impact the plasma CHO level in *S. salar*. Also, suboptimal supplementation of Zn in feed did not alter the plasma CHO level in other fish [63–66].

## 5. Conclusion

To sum up, increased Zn level in feed significantly increased the postprandial plasma Zn level in *S. salar* (8 −9°C). Analyzed markers related to intestinal Zn uptake and transport were not affected by dietary fat level (32%–35% of feed) and n-3 LC-PUFA (2%−3.2% EPA + DHA of feed). Since dietary fat and n-3 LC-PUFA affect whole-body Zn content in Atlantic salmon, further study is needed to explore how these components influence other Zn metabolism processes, such as retention and excretion. Additionally, markers related to intestinal fat transport were unaffected by Zn levels ranging from 120 to 200 mg kg^−1^ in the feed.

## Figures and Tables

**Table 1:** Ingredient and analysis compositions of the experimental diets.

	HFHZ	HFLZ	LFHZ	LFLZ	HPUHZ
Ingrediens (%)					
Fish meal	8.30	8.30	8.30	8.30	8.30
Soy protein concentrate	20.00	20.00	20.00	20.00	20.00
Wheat gluten	10.27	10.27	17.50	17.50	10.27
Guar meal	11.00	11.00	7.24	7.24	11.00
Wheat	13.32	13.36	12.54	12.54	12.57
Fish oil^1^	9.11	9.11	8.29	8.29	15.75
Rapeseed oil, crude	22.19	22.18	20.08	20.08	15.59
Lecithin, dry	0.53	0.53	0.54	0.54	0.55
Mono-calcium phosphate	2.12	2.12	2.38	2.40	2.64
Zink sulphate, 36%	0.04	0.02	0.04	0.02	0.04
Vitamine and mineral premix	0.71	0.71	0.76	0.76	0.71
Technical feed additives	0.55	0.55	0.50	0.50	0.55
Crystalline amino acids	1.40	1.40	1.32	1.32	1.48
Lucantin pink CWD 10%, BASF	0.05	0.05	0.05	0.05	0.05
Cholesterol	0.05	0.05	0.05	0.05	—
Water change	0.38	0.37	0.41	0.41	0.51
Sum	100.00	100.00	100.00	100.00	100.00
Analyzed proximate composition (%), WM					
Moisture	6.3	7.8	7.1	6.8	7.3
Protein	34.8	34.5	38.3	38.5	34.9
Fat	34.7	34.2	31.5	32.3	34.3
Ash	5.1	4.9	5.1	5.0	5.4
Analysed mineral composition (mg kg^−1^), WM					
Zn	204.9	126.6	191.7	129.1	184.3
Fe	204.2	211.1	211.7	218.1	245.3
Mn	46.2	52.7	60.8	54.6	59.8
Cu	6.3	6.0	6.5	6.5	7.0
Se	0.8	0.8	0.8	0.8	0.8
Analysed fatty acid composition (% of total fatty acid)					
Σ SFA	16.64	16.43	16.57	16.75	21.16
14:0	1.59	1.53	1.59	1.65	2.49
16:0	9.01	8.94	9.08	9.22	11.27
18:0	2.85	2.80	2.81	2.81	2.88
20:0	0.60	0.59	0.58	0.57	0.53
22:0	2.58	2.57	2.52	2.50	3.99
Σ MUFA	48.00	48.27	47.91	47.83	41.52
16:1 n-7	0.07	0.07	0.07	0.07	0.11
18:1 n-9	41.58	41.85	41.54	41.55	34.67
18:1 n-7	2.59	2.60	2.59	2.59	2.42
20:1 n-9	2.45	2.45	2.41	2.40	2.97
20:1 n-7	0.08	0.08	0.08	0.08	0.11
22:1 n-9	0.77	0.77	0.76	0.71	0.62
24:1 n-9	0.44	0.45	0.45	0.43	0.61
Σ PUFA	30.47	30.49	30.88	30.77	30.78
Σ n-3	13.68	13.79	13.73	13.64	17.26
18:3 n-3 (ALA)	5.95	6.00	6.00	6.01	4.87
20:4 n-3	0.86	0.86	0.85	0.87	1.39
20:5 n-3 (EPA)	2.54	2.53	2.51	2.48	4.02
22:5 n-3	0.26	0.26	0.26	0.26	0.41
22:6 n-3 (DHA)	4.07	4.14	4.12	4.03	6.56
Σ n-6	16.79	16.70	17.15	17.13	13.52
18:2 n-6 (LA)	16.45	16.37	16.82	16.79	13.02
20:2 n-6	0.18	0.17	0.18	0.19	0.25
20:4 n-6 (ARA)	0.16	0.15	0.16	0.15	0.25
Sum other FAs	4.79	4.79	4.53	4.54	6.33
Sum	99.90	99.98	99.89	99.90	99.78
EPA + DHA, mg g^−1^	19.28	20.88	17.55	19.20	32.33

*Note:* Fish oil^1^: contained 20% EPA + DHA; technical feed additives: antioxidants and fat sealers.

Abbreviations: ALA, alpha-linolenic acid; ARA, arachidonic acid; Cu, copper; DHA, docosahexaenoic acid; EPA, eicosapentaenoic acid; Fe, iron; LA, linoleic acid; Mn, manganese; Se, selenium; WM, wet matter; Zn, zinc.

**Table 2:** Primers of candidate genes for qPCR analysis.

Gene	Primer sequence (5′–3′)	Accession no.^a^	Efficiency (%)	Production (bps)	Tm (°C)
House-keeping genes					

*β*-Actin	F: CCAAAGCCAACAGGGAGAA	BG933897	113.4	91	57.56
	R: AGGGACAACACTGCCTGGAT				61.14

ef-1α	F: TGCCCCTCCAGGATGTCTAC	AF321836	100.3	57	60.69
	R: CACGGCCCACAGGTACTG				60.05

Zinc transport genes					

zip4	F: GAGCCTCCTAGTCCTCACACT	XM_045709302.1	110.6	158	60.34
	R: GTCATTTTGAGGAAATCGTGTATCA				57.93

mta	F: TGCAAGGGCAAGACTTGTGA	NM_001123677.1	104.6	87	60.11
	R: ACGTCAGTCATAGGGAATGGAC				59.57

mtb	F: CTCTTGCAACTGCGGTGGAT	NM_001123669.1	106.6	76	60.96
	R: GCAGGGGCAGCAACTTTTC				60.01

znt1	F: GTATCCTGCTCTACACCACCTA	XM_014143981.2	93.2	148	58.44
	R: CAGATGTGCAGTTCGTGGA				57.78

mtf1	F: AGTTTTTCCACAACAAAAGGGC	XM_014200437.2	115.7	169	58.39
	R: AGAGCTGTTGCTATGGTGGAC				60.07

Lipid transport and metabolism genes					

npc1l1	F: CCCGTCATGAGCCAGGATAC	XM_014171081.2	107.9	162	59.97
	R: GCATGGGGCAGACCTTTTTG				60.04

cd36	F: ACCCCCAGCAGTCACATTATT	XM_014153607.2	100.5	131	59.36
	R: GTATGTAGGTCCCAGCAGCA				59.17

fatp4	F: TCTGGAACACATGACAAGCC	XM_014125609	152.0	170	58.10
	R: GCGAACAAGTTGTGTCCTTCC				60.00

fatp6	F: ACAAACTGCAACCCGCTCTA	XM_045693823.1	115.4	156	59.89
	R: CCACCGTCTCAGTGAACCAA				59.89

fabp2	F: GGATTATGCCTCGACTGCCA	BT048647	115.9	149	59.89
	R: GCCACTCTGGGGAATTGCTA				59.74

dgat1	F: CGGTAACGGAATGGTGCGT	XM_014124329.1	100.1	148	60.74
	R: CAGCCTCTGACATCAATTGCCT				60.94

mtp	F: TGATCATTGTAAAATGTGTGCCTTT	XM_014195517.2	104.7	124	58.24
	R: ACAGCTAGCAAGTTAGCCTC				57.32

apob	F: TGGGCTTGACTGGCAAGATT	X81856	116.0	101	59.89
	R: TCCCTCATCTTGGCGTTTCT				59.02

cpt1	F: TAAGAGGCCGTGGACCAATC	XM_045708590.1	95.5	195	59.46
	R: ATTGCGCTGAGCACATTGGA				60.96

mgat2	F: CGAGTGCAAGCTCTGCAAGG	XM_014205344	113.4	75	61.91
	R: GAGGTCGGGCAAGATGAAGT				59.75

apoa4	F: TGAAGGTGTTGGTGGTGCTT	XM_014201582	126.1	131	60.03
	R: TGTTGCCTTGGCGACATAGT				59.96

^a^GenBank (http://www.ncbi.nlm.nih.gov/).

**Table 3:** Growth performance of *S. salar* fed with different diets in trial 1 and trial 2.

	HFHZ	HFLZ	LFHZ	LFLZ	HPUHZ	*p* Value		
Trial 1	—	—	—	—	—	Zn	Fat	Zn × Fat
IW	741.9 ± 163.7	738.6 ± 121.4	700.5 ± 150.0	694.2 ± 173.6	—	0.93	0.44	0.98
ICF	1.2 ± 0.1	1.2 ± 0.1	1.2 ± 0.1	1.2 ± 0.1	—	0.72	0.44	0.37
FW	888.2 ± 226.8	884 ± 180.3	828.7 ± 190.5	849.5 ± 263.0	—	0.90	0.51	0.86
FCF	1.0 ± 0.1	1.0 ± 0.1	1.0 ± 0.1	1.0 ± 0.1	—	0.71	0.31	0.47
WG	19.6 ± 2.1	19.7 ± 2.4	18.5 ± 4.8	22.2 ± 2.7	—	0.45	0.78	0.48
SR	100	100	100	100	—	—	—	—
Trial 2	—	—	—	—	—	PUFA		Zn
IW	664.3 ± 97.8	663.7 ± 90	—	—	673.0 ± 85.1	0.70		0.98
ICF	1.2 ± 0.1	1.2 ± 0.1	—	—	1.2 ± 0.1	0.83		0.96
FW	925.7 ± 160.6	955.4 ± 169.7	—	—	975.5 ± 138	0.33		0.59
FCF	1.2 ± 0.1	1.2 ± 0.1	—	—	1.2 ± 0.1	0.06		0.16
WG	39.6 ± 4.0	43.7 ± 3.8	—	—	44.9 ± 3.8	0.11		0.27
SR	94.7 ± 6.1	94.7 ± 9.2	—	—	98.7 ± 2.3	0.35		0.99

Abbreviations: FCF, final condition factor; FW, final weight; ICF, initial condition factor; IW, initial weight; PUFA, polyunsaturated fatty acids; SR, survival rate; WG, weight gain; Zn, zinc.

## Data Availability

Experiment data are available from the corresponding author based on reasonable requests.

## Supporting Information

Supporting Information Table S1. Number of fish without pellets in their stomachs during the postprandial sampling. Table S2. Postprandial plasma mineral and lipids of *S. salar* fed with different diets (trial 1). Table S3. Postprandial plasma minerals and lipids of *S. salar* fed with different diets (trial 2). Table S4 Postprandial intestinal mRNA expression related to Zn and lipid uptake and transport in *S. salar* fed with different diets (trial 1). Table S5. Postprandial intestinal mRNA expression related to Zn and lipid uptake and transport in *S. salar* fed with different diets (trial 2).
