# Supplementary material for: The Interaction Between Dietary Fat Level, n-3 LC-PUFA, and Zinc on Their Postprandial Absorption Kinetics in Atlantic Salmon (Salmo salar)
Source: Aquac Nutr. 2024 Dec 28;2024:6173690. doi: 10.1155/anu/6173690 (PMC11699991; doi:10.1155/anu/6173690)
Supplement: Supporting Information — Table S1. Number of fish without pellets in their stomachs during the postprandial sampling. Table S2. Postprandial plasma mineral and lipids of S. salar fed with different diets (trial 1). Table S3. Postprandial plasma minerals and lipids of S. salar fed with different diets (trial 2). Table S4 Postprandial intestinal mRNA expression related to Zn and lipid uptake and transport in S. salar fed with different diets (trial 1). Table S5. Postprandial intestinal mRNA expression related to Zn and lipid uptake and transport in S. salar fed with different diets (trial 2). [file 6173690.f1.docx]

Supplementary table 1: Number of fish without pellets in their stomachs during the postprandial sampling.

| Trial 1 |  |  |  |  |  |  |  |  |
| --- | --- | --- | --- | --- | --- | --- | --- | --- |
| **Diets** | **Tank** | **2h** | **4h** | **8h** | **14h** | **24h** | **36h** |  |
| **HFHZ** | 1 | 0 | 0 | 0 | 1 | 0 | 0 |  |
|  | 2 | 0 | 0 | 0 | 0 | 0 | 0 |  |
| **HFLZ** | 3 | 0 | 1 | 0 | 1 | 0 | 0 |  |
|  | 4 | 0 | 0 | 2 | 0 | 0 | 0 |  |
| **LFHZ** | 5 | 0 | 0 | 0 | 0 | 0 | 1 |  |
|  | 6 | 1 | 1 | 1 | 0 | 0 | 0 |  |
| **LFLZ** | 7 | 1 | 0 | 0 | 0 | 0 | 1 |  |
|  | 8 | 0 | 1 | 0 | 0 | 0 | 0 |  |
|  |  |  |  |  |  |  |  |  |
| Trial 2 |  |  |  |  |  |  |  |  |
| **Diets** | **tank** | **4h** | **6h** | **10h** | **14h** | **26h** | **32h** | **38h** |
| **HFHZ** | 1 | 0 | 0 | 0 | 0 | 0 | 0 | 0 |
|  | 4 | 0 | 0 | 0 | 0 | 0 | 0 | 0 |
|  | 7 | 0 | 0 | 0 | 0 | 0 | 0 | 0 |
| **HFLZ** | 2 | 0 | 0 | 0 | 0 | 0 | 0 | 0 |
|  | 5 | 0 | 0 | 0 | 0 | 0 | 0 | 0 |
|  | 8 | 0 | 0 | 0 | 0 | 0 | 0 | 0 |
| **HPUHZ** | 3 | 0 | 0 | 0 | 1 | 0 | 0 | 0 |
|  | 6 | 0 | 0 | 0 | 0 | 1 | 0 | 0 |
|  | 9 | 0 | 0 | 0 | 0 | 0 | 0 | 0 |

5 fish for each tank in trial 1, and 3 fish for each tank in trial 2.

Supplementary table 2: Postprandial plasma mineral and lipids of *Salmo salar* fed with different diets (trial 1).

|  | **HFHZ** | **HFLZ** | **LFHZ** | **LFLZ** |
| --- | --- | --- | --- | --- |
| **Zn** | | | | |
| 0h | 238.2±37.3 | 201.8±33.4 | 235.5±42.7 | 181.5±23.8 |
| 2h | 249.1±36.6 | 194.5±26.0 | 237.1±44.2 | 174.9±53.5 |
| 4h | 242.4±24.3 | 183.6±34.3 | 230.5±38.8 | 202.4±36.6 |
| 8h | 246.8±23.4 | 186.6±35.9 | 244.8±41.3 | 201.8±30.0 |
| 14h | 235.7±40.7 | 191.8±20.0 | 236.1±53.4 | 207.6±19.2 |
| 24h | 267.5±43.4 | 211.0±38.3 | 264.8±32.0 | 208.1±29.8 |
| 36h | 226.7±40.2 | 207.1±16.2 | 247.7±44.9 | 191.2±33.7 |
| **p value** | | | | |
| Time | | | 0.10 | |
| Fat | | | 0.66 | |
| Zn | | | **<0.01** | |
| Time x Fat | | | 0.32 | |
| Time x Zn | | | 0.67 | |
| Fat x Zn | | | 0.82 | |
| Time x Fat x Zn | | | 0.25 | |
|  | | |  | |
| **CHO** | | | | |
| 0h | 6.4±1.3 | 6.9±1.0 | 6.9±0.8 | 5.9±0.8 |
| 2h | 7.3±0.9 | 6.9±0.8 | 7.3±0.8 | 6.7±1.8 |
| 4h | 7.1±0.8 | 6.4±1.2 | 6.7±0.9 | 6.9±0.7 |
| 8h | 7.0±0.7 | 6.7±1.3 | 7.1±0.8 | 7.2±0.4 |
| 14h | 6.9±1.1 | 6.5±1.1 | 6.4±1.9 | 6.3±2.0 |
| 24h | 6.5±0.9 | 7.2±1.3 | 7.2±0.6 | 7.1±1.0 |
| 36h | 6.3±1.4 | 7.2±0.8 | 6.6±1.2 | 6.5±1.3 |
| **p value** | | | | |
| Time | | | 0.69 | |
| Fat | | | 0.82 | |
| Zn | | | 0.87 | |
| Time x Fat | | | 0.71 | |
| Time x Zn | | | 0.08 | |
| Fat x Zn | | | 0.50 | |
| Time x Fat x Zn | | | 0.51 | |
|  | | |  | |
| **TAG** | | | | |
| 0h | 1.3±0.3 | 1.5±0.3 | 1.2±0.2 | 1.3±0.5 |
| 2h | 1.3±0.4 | 1.4±0.3 | 1.4±0.4 | 1.3±0.4 |
| 4h | 1.3±0.2 | 1.4±0.2 | 1.4±0.3 | 1.4±0.3 |
| 8h | 2.0±0.4 | 1.6±0.3 | 1.6±0.3 | 1.6±0.4 |
| 14h | 1.8±0.7 | 2.0±0.8 | 2.3±0.6 | 1.9±0.3 |
| 24h | 2.4±1.0 | 3.2±1.5 | 2.3±0.6 | 2.6±0.9 |
| 36h | 2.3±0.9 | 2.3±1.3 | 2.6±1.8 | 2.2±0.8 |
| **p value** | | | | |
| Time | | | **<0.01** | |
| Fat | | | 0.61 | |
| Zn | | | 0.57 | |
| Time x Fat | | | 0.98 | |
| Time x Zn | | | 0.68 | |
| Fat x Zn | | | 0.22 | |
| Time x Fat x Zn | | | 0.13 | |
|  | | |  | |
| **HDL** | | | | |
| 0h | 2.5±0.5 | 2.7±0.4 | 2.8±0.4 | 2.3±0.5 |
| 2h | 2.3±1.3 | 2.8±0.4 | 2.9±0.4 | 2.6±0.9 |
| 4h | 2.9±0.4 | 2.5±0.6 | 2.7±0.5 | 2.8±0.2 |
| 8h | 3.0±0.4 | 2.8±0.7 | 3.0±0.6 | 3.1±0.1 |
| 14h | 3.0±0.6 | 2.6±0.6 | 2.7±1.0 | 2.7±0.9 |
| 24h | 2.7±0.5 | 2.9±0.5 | 3.1±0.2 | 2.9±0.5 |
| 36h | 2.4±0.6 | 3.0±0.3 | 2.6±0.6 | 2.5±0.6 |
| **p value** | | | | |
| Time | | | 0.74 | |
| Fat | | | 0.64 | |
| Zn | | | 0.81 | |
| Time x Fat | | | 0.71 | |
| Time x Zn | | | 0.24 | |
| Fat x Zn | | | 0.23 | |
| Time x Fat x Zn | | | 0.71 | |
|  | | |  | |
| **LDL** | | | | |
| 0h | 0.36±0.09 | 0.35±0.13 | 0.42±0.11 | 0.36±0.10 |
| 2h | 0.40±0.14 | 0.38±0.05 | 0.39±0.08 | 0.33±0.14 |
| 4h | 0.37±0.14 | 0.36±0.07 | 0.40±0.09 | 0.32±0.09 |
| 8h | 0.40±0.15 | 0.33±0.09 | 0.43±0.16 | 0.35±0.07 |
| 14h | 0.31±0.08 | 0.38±0.13 | 0.34±0.09 | 0.30±0.12 |
| 24h | 0.30±0.11 | 0.31±0.07 | 0.33±0.10 | 0.28±0.11 |
| 36h | 0.35±0.13 | 0.26±0.08 | 0.33±0.12 | 0.28±0.09 |
| **p value** | | | | |
| Time | | | **<0.01** | |
| Fat | | | 0.99 | |
| Zn | | | 0.09 | |
| Time x Fat | | | 0.83 | |
| Time x Zn | | | 0.73 | |
| Fat x Zn | | | 0.29 | |
| Time x Fat x Zn | | | 0.35 | |
|  | | |  | |
| **TP** | | | | |
| 0h | 29.9±3.2 | 31.8±2.8 | 33.0±4.9 | 28.6±2.3 |
| 2h | 34.6±2.7 | 33.7±2.7 | 34.1±4.1 | 31.8±6.3 |
| 4h | 34.2±3.1 | 31.7±2.8 | 32.5±2.7 | 33.6±3.6 |
| 8h | 35.0±3.1 | 33.2±4.5 | 34.0±3.8 | 34.8±1.6 |
| 14h | 35.4±5.7 | 33.4±3.0 | 33.6±6.2 | 32.1±8.9 |
| 24h | 34.8±3.9 | 35.9±4.4 | 35.4±2.2 | 35.0±5.0 |
| 36h | 30.3±4.6 | 32.0±2.7 | 30.4±4.2 | 30.9±4.7 |
| **p value** | | | | |
| Time | | | 0.65 | |
| Fat | | | 0.51 | |
| Zn | | | 0.49 | |
| Time x Fat | | | 0.82 | |
| Time x Zn | | | 0.19 | |
| Fat x Zn | | | 0.74 | |
| Time x Fat x Zn | | | 0.78 | |
|  | | |  | |
| **Mn** | | | | |
| 0h | 1.4±0.4 | 2.1±1.0 | 1.6±0.5 | 1.2±0.5 |
| 2h | 1.6±0.5 | 1.6±0.4 | 1.7±0.7 | 1.9±0.8 |
| 4h | 2.9±0.8 | 2.2±0.5 | 2.2±0.8 | 2.2±0.8 |
| 8h | 2.9±1.3 | 3.3±1.6 | 3.2±0.9 | 3.3±1.0 |
| 14h | 3.3±1.4 | 2.7±1.4 | 2.3±1.2 | 3.2±1.2 |
| 24h | 2.1±1.0 | 2.4±0.8 | 2.3±0.9 | 3.0±1.3 |
| 36h | 1.4±0.3 | 1.8±0.6 | 1.6±0.7 | 2.1±1.0 |
| **p value** | | | | |
| Time | | | 0.81 | |
| Fat | | | 0.86 | |
| Zn | | | 0.40 | |
| Time x Fat | | | 0.14 | |
| Time x Zn | | | 0.21 | |
| Fat x Zn | | | 0.48 | |
| Time x Fat x Zn | | | 0.41 | |
|  | | |  | |
| **Fe** | | | | |
| 0h | 13.6±4.5 | 16.8±5.1 | 13.9±4.8 | 15.0±4.8 |
| 2h | 13.4±3.7 | 13.7±2.0 | 13.7±4.7 | 17.2±6.1 |
| 4h | 16.8±5.9 | 16.5±6.0 | 17.0±8.6 | 13.8±4.8 |
| 8h | 19.2±7.7 | 17.3±8.9 | 20.5±4.2 | 19.9±4.8 |
| 14h | 20.2±15.0 | 22.6±11.6 | 20.9±7.7 | 14.2±3.6 |
| 24h | 19.6±7.0 | 23.0±12.2 | 23.1±6.3 | 20.7±5.5 |
| 36h | 22.0±6.7 | 16.4±8.0 | 18.8±2.3 | 21.0±10.8 |
| **p value** | | | | |
| Time | | | **<0.01** | |
| Fat | | | 0.99 | |
| Zn | | | 0.85 | |
| Time x Fat | | | 0.74 | |
| Time x Zn | | | 0.27 | |
| Fat x Zn | | | 0.69 | |
| Time x Fat x Zn | | | 0.38 | |
|  | | | | |
| **Cu** | | | | |
| 0h | 6.0±1.8 | 5.5±1.4 | 6.1±1.8 | 6.3±2.0 |
| 2h | 8.2±1.9 | 7.0±2.3 | 7.7±1.8 | 7.9±2.1 |
| 4h | 6.1±1.6 | 6.4±2.0 | 8.3±1.7 | 8.1±2.1 |
| 8h | 7.3±2.0 | 6.3±2.3 | 7.5±2.1 | 6.8±2.2 |
| 14h | 7.2±2.4 | 7.4±1.8 | 6.5±2.0 | 6.9±2.1 |
| 24h | 6.3±2.2 | 6.5±2.0 | 7.9±2.5 | 7.4±1.8 |
| 36h | 9.0±1.8 | 7.8±1.7 | 7.5±1.6 | 6.7±2.0 |
| **p value** |  |  |  |  |
| Time | | | **0.05** | |
| Fat | | | 0.32 | |
| Zn | | | 0.29 | |
| Time x Fat | | | **0.05** | |
| Time x Zn | | | 0.54 | |
| Fat x Zn | | | 0.69 | |
| Time x Fat x Zn | | | 0.54 | |
|  |  |  |  |  |
| **Se** |  |  |  |  |
| 0h | 1.7±0.3 | 1.9±0.3 | 1.8±0.3 | 1.4±0.3 |
| 2h | 1.6±0.3 | 1.7±0.3 | 1.6±0.4 | 1.7±0.3 |
| 4h | 1.7±0.2 | 1.5±0.2 | 1.6±0.3 | 1.6±0.3 |
| 8h | 1.7±0.2 | 1.8±0.3 | 1.6±0.3 | 1.8±0.3 |
| 14h | 1.7±0.3 | 1.6±0.3 | 1.6±0.2 | 1.7±0.3 |
| 24h | 1.7±0.2 | 2.0±0.4 | 1.9±0.2 | 1.7±0.3 |
| 36h | 1.6±0.4 | 1.8±0.2 | 1.6±0.3 | 1.7±0.3 |
| **p value** |  |  |  |  |
| Time | | | 0.22 | |
| Fat | | | 0.53 | |
| Zn | | | 0.68 | |
| Time x Fat | | | 0.65 | |
| Time x Zn | | | 0.09 | |
| Fat x Zn | | | 0.54 | |
| Time x Fat x Zn | | | 0.51 | |

The values mean ± SD (n=10).

Supplementary table 3: Postprandial plasma minerals and lipids of *Salmo salar* fed with different diets (trial 2).

|  | **HFHZ** | **HFLZ** | **HPUHZ** |
| --- | --- | --- | --- |
| **Zn** |  |  |  |
| 0h | 210.9±37.7 | 185.3±33.7 | 227.3±43.5 |
| 4h | 224.8±41.4 | 214.4±30.0 | 250.0±41.2 |
| 6h | 211.6±44.3 | 202.3±44.3 | 227.9±43.1 |
| 10h | 257.7±22.8 | 214.4±29.3 | 257.5±42.8 |
| 14h | 267.0±39.1 | 240.2±21.7 | 245.5±19.7 |
| 26h | 236.4±15.2 | 237.7±23.5 | 268.7±56.6 |
| 32h | 249.5±23.4 | 185.3±45.8 | 245.7±36.8 |
| 38h | 214.2±30.5 | 209.3±25.8 | 257.8±44.1 |
| **p value (HFHZ vs HPUHZ)** | |  |  |
| PUFA x Time | | 0.46 | |
| Time | | 0.08 | |
| PUFA | | 0.41 | |
| **p value (HFHZ vs HFLZ)** | |  | |
| Zn x Time | | 0.99 | |
| Time | | 0.29 | |
| Zn | | 0.20 | |
|  |  |  |  |
| **CHO** |  |  |  |
| 0h | 7.8±1.3 | 8.2±1.6 | 7.9±1.9 |
| 4h | 7.5±1.8 | 9.0±1.1 | 8.1±1.2 |
| 6h | 7.1±1.1 | 7.9±1.6 | 7.4±1.4 |
| 10h | 7.1±2.2 | 8.2±0.8 | 7.5±0.8 |
| 14h | 7.8±1.0 | 8.4±0.9 | 7.2±1.1 |
| 26h | 7.5±0.9 | 8.8±0.7 | 8.3±1.5 |
| 32h | 9.4±1.4 | 8.7±1.8 | 9.1±1.4 |
| 38h | 7.8±1.8 | 9.7±1.0 | 9.0±1.7 |
| **p value (HFHZ vs HPUHZ)** | |  | |
| PUFA x Time | | 0.35 | |
| Time | | **<0.01** | |
| PUFA | | 0.64 | |
| **p value (HFHZ vs HFLZ)** | |  | |
| Zn x Time | | 0.73 | |
| Time | | **0.01** | |
| Zn | | 0.19 | |
|  |  |  |  |
| **TAG** |  |  |  |
| 0h | 1.1±0.3 | 1.1±0.3 | 1.0±0.2 |
| 4h | 1.0±0.2 | 1.3±0.5 | 1.1±0.2 |
| 6h | 0.9±0.3 | 1.3±0.7 | 1.0±0.3 |
| 10h | 1.7±0.5 | 1.5±0.3 | 1.5±0.5 |
| 14h | 1.6±0.4 | 1.6±0.4 | 1.7±0.3 |
| 26h | 1.8±0.8 | 2.2±0.8 | 1.6±0.3 |
| 32h | 2.2±0.9 | 2.8±1.2 | 2.0±0.7 |
| 38h | 1.6±0.6 | 1.8±0.6 | 1.5±0.6 |
| **p value (HFHZ vs HPUHZ)** | |  | |
| PUFA x Time | | 0.42 | |
| Time | | **<0.01** | |
| PUFA | | 0.43 | |
| **p value (HFHZ vs HFLZ)** | |  | |
| Zn x Time | | 0.40 | |
| Time | | **<0.01** | |
| Zn | | 0.25 | |
|  |  |  |  |
| **HDL** |  |  |  |
| 0h | 2.7±0.4 | 2.9±0.6 | 2.5±0.7 |
| 4h | 2.6±0.5 | 3.3±0.6 | 2.7±0.5 |
| 6h | 2.8±0.6 | 3.0±0.8 | 2.6±0.6 |
| 10h | 2.8±1.0 | 2.9±1.1 | 2.6±0.4 |
| 14h | 3.1±0.5 | 3.2±0.4 | 2.6±0.4 |
| 26h | 3.1±0.4 | 3.5±0.5 | 3.1±0.7 |
| 32h | 3.4±0.4 | 2.8±0.7 | 3.1±0.5 |
| 38h | 2.6±0.8 | 3.3±0.3 | 3.1±0.5 |
| **p value (HFHZ vs HPUHZ)** | |  | |
| PUFA x Time | | 0.26 | |
| Time | | **<0.01** | |
| PUFA | | 0.68 | |
| **p value (HFHZ vs HFLZ)** | |  | |
| Zn x Time | | 0.85 | |
| Time | | 0.10 | |
| Zn | | 0.27 | |
|  |  |  |  |
| **LDL** |  |  |  |
| 0h | 0.42±0.22 | 0.48±0.19 | 0.31±0.09 |
| 4h | 0.54±0.27 | 0.48±0.12 | 0.37±0.12 |
| 6h | 0.33±0.14 | 0.32±0.13 | 0.32±0.16 |
| 10h | 0.29±0.08 | 0.28±0.15 | 0.33±0.09 |
| 14h | 0.33±0.05 | 0.33±0.08 | 0.32±0.09 |
| 26h | 0.35±0.11 | 0.45±0.23 | 0.34±0.06 |
| 32h | 0.36±0.18 | 0.39±0.19 | 0.24±0.06 |
| 38h | 0.35±0.12 | 0.41±0.24 | 0.25±0.08 |
| **p value (HFHZ vs HPUHZ)** | |  | |
| PUFA x Time | | 0.95 | |
| Time | | **0.01** | |
| PUFA | | 0.28 | |
| **p value (HFHZ vs HFLZ)** | |  | |
| Zn x Time | | 0.40 | |
| Time | | 0.28 | |
| Zn | | 0.63 | |
|  | |  | |
| **Totalt protein** |  |  |  |
| 0h | 33.7±2.8 | 34.4±5.8 | 33.6±5.5 |
| 4h | 34.3±6.4 | 38.4±3.8 | 36.5±3.8 |
| 6h | 32.2±4.2 | 32.7±3.0 | 31.5±4.5 |
| 10h | 29.5±6.9 | 33.5±2.1 | 31.2±4.1 |
| 14h | 39.7±4.1 | 42.5±2.7 | 37.6±2.9 |
| 26h | 34.1±3.1 | 36.8±2.3 | 37.2±5.4 |
| 32h | 35.0±3.3 | 33.1±2.4 | 35.0±2.7 |
| 38h | 32.3±3.6 | 35.4±2.0 | 34.9±4.8 |
| **p value (HFHZ vs HPUHZ)** | |  | |
| PUFA x Time | | 0.44 | |
| Time | | 0.29 | |
| PUFA | | 0.67 | |
| **p value (HFHZ vs HFLZ)** | |  | |
| Zn x Time | | 0.92 | |
| Time | | 0.86 | |
| Zn | | 0.26 | |
|  |  |  |  |
| **Mn** |  |  |  |
| 0h | 1.4±0.6 | 1.9±0.7 | 2.2±0.8 |
| 4h | 1.3±0.4 | 2.5±0.6 | 2.7±0.9 |
| 6h | 2.5±0.7 | 3.0±1.2 | 3.1±1.1 |
| 10h | 3.4±1.3 | 3.5±0.9 | 3.6±0.9 |
| 14h | 3.3±0.8 | 3.7±0.6 | 4.5±0.8 |
| 26h | 2.0±0.3 | 3.2±1.3 | 3.7±1.4 |
| 32h | 2.4±0.8 | 2.0±0.9 | 2.9±0.9 |
| 38h | 1.5±0.6 | 2.3±0.7 | 2.7±1.2 |
| **p value (HFHZ vs HPUHZ)** | |  | |
| PUFA x Time | | 0.59 | |
| Time | | 0.60 | |
| PUFA | | **0.01** | |
| **p value (HFHZ vs HFLZ)** | |  | |
| Zn x Time | | 0.97 | |
| Time | | 0.81 | |
| Zn | | **0.04** | |
|  |  |  |  |
| **Fe** |  |  |  |
| 0h | 9.1±2.4 | 11.2±5.6 | 14.2±6.6 |
| 4h | 13.3±8.7 | 12.4±3.7 | 11.2±2.1 |
| 6h | 9.1±4.2 | 10.1±2.3 | 9.5±3.5 |
| 10h | 15.0±4.6 | 17.1±7.9 | 13.6±5.3 |
| 14h | 15.9±9.3 | 14.7±3.2 | 16.2±7.1 |
| 26h | 14.4±4.8 | 18.5±8.5 | 13.1±3.8 |
| 32h | 14.8±6.3 | 13.4±4.2 | 11.8±2.9 |
| 38h | 12.9±3.6 | 14.9±3.1 | 14.7±4.2 |
| **p value (HFHZ vs HPUHZ)** | |  |  |
| PUFA x Time | | 0.41 | |
| Time | | 0.09 | |
| PUFA | | 0.96 | |
| **p value (HFHZ vs HFLZ)** | |  |  |
| Zn x Time | | 0.77 | |
| Time | | **0.01** | |
| Zn | | 0.38 | |
|  |  |  |  |
| **Cu** |  |  |  |
| 0h | 9.1±1.0 | 8.7±1.3 | 9.8±2.2 |
| 4h | 10.3±1.5 | 10.4±1.2 | 10.3±1.0 |
| 6h | 9.4±1.6 | 10.3±1.3 | 9.4±1.6 |
| 10h | 9.2±2.0 | 10.7±2.0 | 9.9±1.4 |
| 14h | 9.8±1.1 | 10.2±0.7 | 9.5±0.7 |
| 26h | 10.5±1.1 | 10.2±1.2 | 10.5±1.6 |
| 32h | 10.1±1.1 | 9.4±0.9 | 10.4±0.8 |
| 38h | 9.4±1.0 | 10.4±0.9 | 10.1±1.3 |
| **p value (HFHZ vs HPUHZ)** | |  |  |
| PUFA x Time | | 0.85 | |
| Time | | 0.14 | |
| PUFA | | 0.67 | |
| **p value (HFHZ vs HFLZ)** | |  | |
| Zn x Time | | 0.90 | |
| Time | | 0.36 | |
| Zn | | 0.51 | |
|  |  |  |  |
| **Se** |  |  |  |
| 0h | 1.6±0.2 | 1.7±0.2 | 1.7±0.2 |
| 4h | 1.8±0.3 | 1.9±0.3 | 1.8±0.2 |
| 6h | 1.7±0.3 | 1.7±0.3 | 1.7±0.3 |
| 10h | 1.8±0.5 | 1.8±0.2 | 1.8±0.2 |
| 14h | 1.9±0.2 | 2.1±0.1 | 1.8±0.1 |
| 26h | 1.8±0.2 | 1.9±0.1 | 2.0±0.2 |
| 32h | 1.9±0.1 | 1.6±0.3 | 1.6±0.2 |
| 38h | 1.6±0.3 | 1.8±0.3 | 1.7±0.3 |
| **p value (HFHZ vs HPUHZ)** | |  |  |
| PUFA x Time | | 0.91 | |
| Time | | 0.78 | |
| PUFA | | 0.97 | |
| **p value (HFHZ vs HFLZ)** | |  | |
| Zn x Time | | 0.77 | |
| Time | | 0.66 | |
| Zn | | 0.49 | |

The values mean ± SD (n=9).

Supplementary table 4: Postprandial intestinal mRNA expression related to Zn and lipid uptake and transport in *Salmo salar* fed with different diets (trial 1).

|  | **HFHZ** | | **HFLZ** | **LFHZ** | **LFLZ** |
| --- | --- | --- | --- | --- | --- |
| **apoa4** | | | | | |
| 2h | 1.27±1.13 | | 0.69±0.35 | 1.17±0.42 | 0.80±0.58 |
| 14h | 1.09±0.40 | | 0.74±0.21 | 1.39±1.08 | 0.67±1.05 |
| 24h | 0.95±0.69 | | 0.55±0.20 | 0.99±0.59 | 0.62±0.37 |
| **p value** | | | | | |
| Time | | | | 0.20 | |
| Fat | | | | 0.75 | |
| Zn | | | | 0.08 | |
| Time x Fat | | | | 0.89 | |
| Time x Zn | | | | 0.88 | |
| Fat x Zn | | | | 0.96 | |
| Time x Fat x Zn | | | | 0.51 | |
|  | | | |  | |
| **apob** | | | | | |
| 2h | 0.34±0.33 | | 0.76±0.98 | 0.10±0.08 | 1.39±1.73 |
| 14h | 0.30±0.29 | | 0.34±0.34 | 0.32±0.24 | 0.23±0.17 |
| 24h | 0.36±0.43 | | 0.22±0.17 | 0.27±0.17 | 0.26±0.22 |
| **p value** | | | | | |
| Time | | | | **0.03** | |
| Fat | | | | 0.71 | |
| Zn | | | | 0.31 | |
| Time x Fat | | | | 0.81 | |
| Time x Zn | | | | **0.01** | |
| Fat x Zn | | | | 0.50 | |
| Time x Fat x Zn | | | | 0.35 | |
|  | | | |  | |
| **cd36** | | | | | |
| 2h | 2.29±1.26 | | 2.11±0.75 | 2.05±0.93 | 2.05±0.98 |
| 14h | 1.60±0.37 | | 1.62±0.36 | 2.04±0.85 | 1.93±1.02 |
| 24h | 1.26±0.43 | | 1.10±0.35 | 1.54±0.70 | 1.11±0.43 |
| **p value** | | | | | |
| Time | | | | **<0.01** | |
| Fat | | | | 0.65 | |
| Zn | | | | 0.67 | |
| Time x Fat | | | | 0.24 | |
| Time x Zn | | | | 0.76 | |
| Fat x Zn | | | | 0.95 | |
| Time x Fat x Zn | | | | 0.86 | |
|  | | | |  | |
| **cpt1** | | | | | |
| 2h | 2.10±1.34 | | 1.67±0.86 | 1.75±0.67 | 2.44±1.14 |
| 14h | 1.10±0.22 | | 1.06±0.16 | 2.30±1.18 | 2.37±1.45 |
| 24h | 1.60±1.11 | | 1.06±0.18 | 1.66±1.00 | 1.22±0.47 |
| **p value** | | | | | |
| Time | | | | **0.03** | |
| Fat | | | | 0.18 | |
| Zn | | | | 0.81 | |
| Time x Fat | | | | **0.01** | |
| Time x Zn | | | | 0.32 | |
| Fat x Zn | | | | 0.47 | |
| Time x Fat x Zn | | | | 0.47 | |
|  | | | |  | |
| **dgat1** | | | | | |
| 2h | 3.26±2.28 | | 2.27±0.76 | 2.39±1.40 | 2.54±1.54 |
| 14h | 1.54±0.47 | | 1.91±0.51 | 2.84±1.91 | 3.19±1.32 |
| 24h | 1.23±0.75 | | 1.15±0.31 | 1.43±0.84 | 1.19±0.45 |
| **p value** | | | | | |
| Time | | | | **<0.01** | |
| Fat | | | | 0.33 | |
| Zn | | | | 0.83 | |
| Time x Fat | | | | **0.02** | |
| Time x Zn | | | | 0.31 | |
| Fat x Zn | | | | 0.65 | |
| Time x Fat x Zn | | | | 0.40 | |
|  | | | |  | |
| **fabp2** | | | | | |
| 2h | 1.38±0.73 | | 1.49±0.77 | 1.54±0.92 | 1.31±0.92 |
| 14h | 1.29±0.43 | | 1.27±0.62 | 1.53±0.94 | 1.20±1.28 |
| 24h | 1.18±0.82 | | 0.90±0.36 | 1.68±0.90 | 1.39±0.67 |
| **p value** | | | | | |
| Time | | | |  | |
| Fat | | | | 0.64 | |
| Zn | | | | 0.33 | |
| Time x Fat | | | | 0.42 | |
| Time x Zn | | | | 0.28 | |
| Fat x Zn | | | | 0.82 | |
| Time x Fat x Zn | | | | 0.61 | |
|  | | | | 0.87 | |
| **fatp4** | | | | | |
| 2h | 1.08±0.99 | | 0.63±0.73 | 1.36±0.95 | 1.27±0.94 |
| 14h | 0.45±0.35 | | 0.66±0.49 | 1.41±1.43 | 0.54±1.34 |
| 24h | 0.19±0.27 | | 0.14±0.14 | 0.14±0.12 | 0.10±0.15 |
| **p value** | | | | | |
| Time | | | | **<0.01** | |
| Fat | | | | 0.28 | |
| Zn | | | | 0.44 | |
| Time x Fat | | | | 0.26 | |
| Time x Zn | | | | 0.74 | |
| Fat x Zn | | | | 0.66 | |
| Time x Fat x Zn | | | | 0.12 | |
|  | | | |  | |
| **fatp6** | | | | | |
| 2h | 1.31±0.93 | | 1.74±0.86 | 1.63±0.48 | 1.74±0.73 |
| 14h | 1.02±0.64 | | 1.26±0.44 | 1.41±0.49 | 1.59±1.13 |
| 24h | 0.72±0.32 | | 0.54±0.18 | 0.62±0.31 | 0.59±0.33 |
| **p value** | | | | | |
| Time | | | | **<0.01** | |
| Fat | | | | 0.53 | |
| Zn | | | | 0.62 | |
| Time x Fat | | | | 0.39 | |
| Time x Zn | | | | 0.47 | |
| Fat x Zn | | | | 0.98 | |
| Time x Fat x Zn | | | | 0.60 | |
|  | | | | | |
| **mgat2** | | | | | |
| 2h | 2.58±1.91 | | 1.94±0.50 | 2.11±0.79 | 2.49±1.07 |
| 14h | 1.73±0.41 | | 1.96±0.96 | 3.07±1.95 | 2.78±2.37 |
| 24h | 1.17±0.70 | | 0.86±0.19 | 1.54±0.58 | 0.93±0.38 |
| **p value** |  | |  |  |  |
| Time | | | | **<0.01** | |
| Fat | | | | 0.26 | |
| Zn | | | | 0.67 | |
| Time x Fat | | | | 0.08 | |
| Time x Zn | | | | 0.66 | |
| Fat x Zn | | | | 0.88 | |
| Time x Fat x Zn | | | | 0.29 | |
|  |  | |  |  |  |
| **mta** |  | |  |  |  |
| 2h | 1.25±0.50 | | 1.10±0.56 | 0.86±0.62 | 1.05±0.57 |
| 14h | 0.96±1.03 | | 1.04±0.60 | 0.82±0.43 | 1.38±1.05 |
| 24h | 0.93±0.51 | | 0.90±0.39 | 0.93±0.62 | 0.73±0.45 |
| **p value** |  | |  |  |  |
| Time | | | | 0.39 | |
| Fat | | | | 0.80 | |
| Zn | | | | 0.70 | |
| Time x Fat | | | | 0.49 | |
| Time x Zn | | | | 0.37 | |
| Fat x Zn | | | | 0.66 | |
| Time x Fat x Zn | | | | 0.57 | |
|  | |  |  |  |  |
| **mtb** | |  |  |  |  |
| 2h | | 0.91±0.69 | 0.83±0.39 | 0.89±0.37 | 0.56±0.40 |
| 14h | | 0.72±0.67 | 0.60±0.36 | 0.92±0.60 | 0.83±0.46 |
| 24h | | 0.80±0.36 | 0.55±0.17 | 0.66±0.33 | 0.56±0.28 |
| **p value** | |  |  |  |  |
| Time | | | | 0.28 | |
| Fat | | | | 0.87 | |
| Zn | | | | 0.15 | |
| Time x Fat | | | | 0.23 | |
| Time x Zn | | | | 0.90 | |
| Fat x Zn | | | | 0.91 | |
| Time x Fat x Zn | | | | 0.64 | |
|  | | | |  | |
| **mtf1** | |  |  |  |  |
| 2h | | 1.01±0.26 | 1.48±0.26 | 0.97±0.26 | 1.49±0.66 |
| 14h | | 0.64±0.19 | 0.88±0.27 | 0.82±0.32 | 0.86±0.44 |
| 24h | | 0.95±0.38 | 0.94±0.37 | 1.50±1.03 | 1.06±0.32 |
| **p value** | |  |  |  |  |
| Time | | | | **<0.01** | |
| Fat | | | | 0.25 | |
| Zn | | | | 0.26 | |
| Time x Fat | | | | 0.13 | |
| Time x Zn | | | | **0.01** | |
| Fat x Zn | | | | 0.44 | |
| Time x Fat x Zn | | | | 0.59 | |
|  | | | |  | |
| **mtp** | |  |  |  |  |
| 2h | | 2.08±1.14 | 1.50±0.52 | 1.63±0.56 | 1.93±0.75 |
| 14h | | 1.20±0.40 | 1.35±0.64 | 2.44±1.35 | 1.78±1.60 |
| 24h | | 1.28±0.65 | 0.97±0.30 | 1.68±0.75 | 0.98±0.42 |
| **p value** | |  |  |  |  |
| Time | | | | **0.01** | |
| Fat | | | | 0.22 | |
| Zn | | | | 0.35 | |
| Time x Fat | | | | **0.04** | |
| Time x Zn | | | | 0.54 | |
| Fat x Zn | | | | 0.90 | |
| Time x Fat x Zn | | | | 0.06 | |
|  | | | |  | |
| **npc1l1** | |  |  |  |  |
| 2h | | 1.22±0.70 | 0.67±0.22 | 1.07±0.59 | 1.41±0.86 |
| 14h | | 0.66±0.34 | 0.49±0.18 | 0.74±0.45 | 1.12±0.49 |
| 24h | | 1.51±0.96 | 0.80±0.14 | 1.51±0.83 | 1.22±0.37 |
| **p value** | |  |  |  |  |
| Time | | | | **<0.01** | |
| Fat | | | | 0.07 | |
| Zn | | | | 0.21 | |
| Time x Fat | | | | 0.91 | |
| Time x Zn | | | | 0.08 | |
| Fat x Zn | | | | 0.05 | |
| Time x Fat x Zn | | | | 0.69 | |
|  | | | |  | |
| **zip4** | |  |  |  |  |
| 2h | | 0.89±0.9 | 0.72±0.57 | 0.75±0.61 | 0.88±0.78 |
| 14h | | 0.69±0.39 | 0.80±0.58 | 0.77±0.27 | 0.65±0.52 |
| 24h | | 1.74±1.44 | 0.95±0.57 | 1.51±1.05 | 0.86±0.66 |
| **p value** | |  |  |  |  |
| Time | | | | **<0.01** | |
| Fat | | | | 0.95 | |
| Zn | | | | 0.56 | |
| Time x Fat | | | | 0.96 | |
| Time x Zn | | | | 0.06 | |
| Fat x Zn | | | | 0.93 | |
| Time x Fat x Zn | | | | 0.77 | |
|  | | | |  | |
| **znt1** | |  |  |  |  |
| 2h | | 0.72±0.16 | 0.70±0.24 | 0.74±0.28 | 1.06±0.34 |
| 14h | | 0.77±0.44 | 0.64±0.22 | 0.82±0.36 | 0.97±0.36 |
| 24h | | 1.45±1.22 | 0.95±0.23 | 1.13±0.52 | 0.93±0.35 |
| **p value** | |  |  |  |  |
| Time | | | | **0.01** | |
| Fat | | | | 0.54 | |
| Zn | | | | 0.50 | |
| Time x Fat | | | | 0.24 | |
| Time x Zn | | | | 0.07 | |
| Fat x Zn | | | | 0.19 | |
| Time x Fat x Zn | | | | 0.99 | |

The values mean ± SD (n=10).

Supplementary table 5: Postprandial intestinal mRNA expression related to Zn and lipid uptake and transport in *Salmo salar* fed with different diets (trial 2).

|  | | **HFHZ** | **HFLZ** | **HPUHZ** |
| --- | --- | --- | --- | --- |
| **apoa4** | |  |  |  |
| 4 h | | 0.58±0.29 | 0.43±0.11 | 0.47±0.06 |
| 14 h | | 1.20±0.60 | 1.13±0.53 | 0.86±0.41 |
| 26 h | | 0.80±0.41 | 0.80±0.25 | 0.97±0.61 |
| **p value (HFHZ vs HPUHZ)** | | |  |  |
| Time | | | **0.02** | |
| PUFA | | | 0.63 | |
| PUFA x Time | | | 0.32 | |
| **p value (HFHZ vs HFLZ)** | | |  | |
| Time | | | 0.08 | |
| Zn | | | 0.76 | |
| Zn x Time | | | 0.53 | |
|  | |  |  |  |
| **apob** | |  |  |  |
| 4 h | | 1.49±1.19 | 1.09±1.31 | 1.51±1.55 |
| 14 h | | 1.17±0.94 | 0.74±0.56 | 0.65±0.42 |
| 26 h | | 0.28±0.35 | 0.40±0.36 | 0.24±0.13 |
| **p value (HFHZ vs HPUHZ)** | | |  | |
| Time | | | **<0.01** | |
| PUFA | | | 0.61 | |
| PUFA x Time | | | 0.96 | |
| **p value (HFHZ vs HFLZ)** | | |  | |
| Time | | | **<0.01** | |
| Zn | | | 0.43 | |
| Zn x Time | | | 0.34 | |
|  | |  |  |  |
| **cd36** | |  |  |  |
| 4 h | | 3.26±2.45 | 1.54±1.78 | 3.59±3.06 |
| 14 h | | 1.12±1.21 | 1.21±0.85 | 0.64±0.32 |
| 26 h | | 0.87±1.14 | 0.44±0.63 | 0.63±0.60 |
| **p value (HFHZ vs HPUHZ)** | | |  | |
| Time | | | **<0.01** | |
| PUFA | | | 0.82 | |
| PUFA x Time | | | 0.64 | |
| **p value (HFHZ vs HFLZ)** | | |  | |
| Time | | | **<0.01** | |
| Zn | | | 0.18 | |
| Zn x Time | | | 0.25 | |
|  | |  |  |  |
| **cpt1** | |  |  |  |
| 4 h | | 2.58±1.30 | 2.36±0.75 | 2.38±0.53 |
| 14 h | | 0.32±0.31 | 0.26±0.15 | 0.19±0.10 |
| 26 h | | 0.82±0.46 | 0.82±0.46 | 1.02±0.81 |
| **p value (HFHZ vs HPUHZ)** | | |  | |
| Time | | | **<0.01** | |
| PUFA | | | 0.90 | |
| PUFA x Time | | | 0.55 | |
| **p value (HFHZ vs HFLZ)** | | |  | |
| Time | | | **<0.01** | |
| Zn | | | 0.76 | |
| Zn x Time | | | 0.73 | |
|  | |  |  |  |
| **dgat1** | |  |  |  |
| 4 h | | 2.98±2.40 | 1.36±1.64 | 3.50±3.02 |
| 14 h | | 1.03±1.21 | 1.09±0.90 | 0.56±0.34 |
| 26 h | | 1.01±1.28 | 0.49±0.71 | 0.67±0.66 |
| **p value (HFHZ vs HPUHZ)** | | |  | |
| Time | | | **<0.01** | |
| PUFA | | | 0.87 | |
| PUFA x Time | | | 0.48 | |
| **p value (HFHZ vs HFLZ)** | | |  | |
| Time | | | **<0.01** | |
| Zn | | | 0.18 | |
| Zn x Time | | | 0.32 | |
|  | | |  | |
| **fabp2** | |  |  |  |
| 4 h | | 0.96±0.49 | 0.79±0.25 | 1.04±0.31 |
| 14 h | | 1.41±0.71 | 1.69±0.91 | 1.08±0.30 |
| 26 h | | 1.75±1.80 | 2.86±3.59 | 3.00±3.74 |
| **p value (HFHZ vs HPUHZ)** | | |  | |
| Time | | | **0.01** | |
| PUFA | | | 0.74 | |
| PUFA x Time | | | 0.26 | |
| **p value (HFHZ vs HFLZ)** | | |  | |
| Time | | | **<0.01** | |
| Zn | | | 0.63 | |
| Zn x Time | | | 0.23 | |
|  | |  |  |  |
| **fatp4** | |  |  |  |
| 4 h | | 2.63±1.59 | 1.49±1.07 | 2.84±1.90 |
| 14 h | | 0.23±0.33 | 0.12±0.16 | 0.05±0.06 |
| 26 h | | 0.80±0.86 | 0.35±0.37 | 0.59±0.54 |
| **p value (HFHZ vs HPUHZ)** | | |  | |
| Time | | | **<0.01** | |
| PUFA | | | 0.99 | |
| PUFA x Time | | | 0.68 | |
| **p value (HFHZ vs HFLZ)** | | |  | |
| Time | | | **<0.01** | |
| Zn | | | 0.17 | |
| Zn x Time | | | 0.37 | |
|  | |  |  |  |
| **fatp6** | |  |  |  |
| 4 h | | 3.24±2.44 | 1.58±1.78 | 3.67±3.20 |
| 14 h | | 1.20±1.33 | 1.38±1.00 | 0.67±0.35 |
| 26 h | | 0.74±0.79 | 0.44±0.62 | 0.65±0.55 |
| **p value (HFHZ vs HPUHZ)** | | |  |  |
| Time | | | **<0.01** | |
| PUFA | | | 0.92 | |
| PUFA x Time | | | 0.68 | |
| **p value (HFHZ vs HFLZ)** | | |  |  |
| Time | | | **<0.01** | |
| Zn | | | 0.22 | |
| Zn x Time | | | 0.21 | |
|  | |  |  |  |
| **mgat2** | |  |  |  |
| 4 h | | 1.98±0.97 | 1.21±0.36 | 1.70±0.65 |
| 14 h | | 1.80±0.94 | 1.61±0.68 | 1.27±0.30 |
| 26 h | | 1.83±1.72 | 2.08±1.94 | 2.94±2.80 |
| **p value (HFHZ vs HPUHZ)** | | |  |  |
| Time | | | 0.13 | |
| PUFA | | | 0.69 | |
| PUFA x Time | | | 0.25 | |
| **p value (HFHZ vs HFLZ)** | | |  | |
| Time | | | 0.35 | |
| Zn | | | 0.61 | |
| Zn x Time | | | 0.20 | |
|  | |  |  |  |
| **mta** | |  |  |  |
| 4 h | | 2.02±1.31 | 0.66±0.31 | 2.05±0.98 |
| 14 h | | 1.22±0.53 | 0.62±0.58 | 0.78±0.22 |
| 26 h | | 1.62±1.02 | 0.84±0.41 | 1.69±2.01 |
| **p value (HFHZ vs HPUHZ)** | | |  |  |
| Time | | | 0.36 | |
| PUFA | | | 0.79 | |
| PUFA x Time | | | 0.98 | |
| **p value (HFHZ vs HFLZ)** | | |  | |
| Time | | | 0.78 | |
| Zn | | | **0.03** | |
| Zn x Time | | | 0.33 | |
|  |  | |  |  |
| **mtb** |  | |  |  |
| 4 h | 1.16±0.56 | | 0.44±0.20 | 0.81±0.23 |
| 14 h | 1.21±0.92 | | 0.57±0.58 | 0.78±0.44 |
| 26 h | 0.76±0.53 | | 0.37±0.21 | 1.12±1.23 |
| **p value (HFHZ vs HPUHZ)** | | |  | |
| Time | | | 0.80 | |
| PUFA | | | 0.59 | |
| PUFA x Time | | | 0.13 | |
| **p value (HFHZ vs HFLZ)** | | |  | |
| Time | | | 0.31 | |
| Zn | | | **0.02** | |
| Zn x Time | | | 0.38 | |
|  |  | |  |  |
| **mtf1** |  | |  |  |
| 4 h | 3.05±2.31 | | 1.51±1.74 | 3.53±3.11 |
| 14 h | 1.06±1.24 | | 1.19±0.92 | 0.57±0.37 |
| 26 h | 0.45±0.34 | | 0.18±0.21 | 0.51±0.53 |
| **p value (HFHZ vs HPUHZ)** | | |  | |
| Time | | | **<0.01** | |
| PUFA | | | 0.94 | |
| PUFA x Time | | | 0.76 | |
| **p value (HFHZ vs HFLZ)** | | |  | |
| Time | | | **<0.01** | |
| Zn | | | 0.22 | |
| Zn x Time | | | 0.21 | |
|  |  | |  |  |
| **mtp** |  | |  |  |
| 4 h | 2.24±0.88 | | 1.56±0.26 | 1.92±0.18 |
| 14 h | 1.59±0.56 | | 1.57±0.80 | 1.07±0.35 |
| 26 h | 1.14±0.65 | | 1.05±0.54 | 1.12±0.73 |
| **p value (HFHZ vs HPUHZ)** | | |  | |
| Time | | | **<0.01** | |
| PUFA | | | 0.14 | |
| PUFA x Time | | | 0.56 | |
| **p value (HFHZ vs HFLZ)** | | |  | |
| Time | | | **<0.01** | |
| Zn | | | 0.19 | |
| Zn x Time | | | 0.19 | |
|  |  | |  |  |
| **npc1l1** |  | |  |  |
| 4 h | 3.43±1.52 | | 3.21±0.64 | 3.32±0.77 |
| 14 h | 0.87±0.43 | | 1.14±0.36 | 0.82±0.26 |
| 26 h | 0.97±0.36 | | 1.00±0.47 | 1.21±1.06 |
| **p value (HFHZ vs HPUHZ)** | | |  | |
| Time | | | **<0.01** | |
| PUFA | | | 0.94 | |
| PUFA x Time | | | 0.64 | |
| **p value (HFHZ vs HFLZ)** | | |  | |
| Time | | | **<0.01** | |
| Zn | | | 0.85 | |
| Zn x Time | | | 0.73 | |
|  |  | |  |  |
| **zip4** |  | |  |  |
| 4 h | 0.28±0.19 | | 0.30±0.21 | 0.15±0.08 |
| 14 h | 0.61±0.41 | | 1.06±0.41 | 0.33±0.16 |
| 26 h | 0.58±0.35 | | 0.47±0.17 | 0.75±0.75 |
| **p value (HFHZ vs HPUHZ)** | | |  | |
| Time | | | **<0.01** | |
| PUFA | | | 0.61 | |
| PUFA x Time | | | 0.20 | |
| **p value (HFHZ vs HFLZ)** | | |  | |
| Time | | | **0.02** | |
| Zn | | | 0.50 | |
| Zn x Time | | | 0.74 | |
|  |  | |  |  |
| **znt1** |  | |  |  |
| 4 h | 3.20±1.12 | | 2.18±1.14 | 3.27±1.61 |
| 14 h | 1.24±1.04 | | 1.36±0.66 | 0.74±0.22 |
| 26 h | 0.65±0.46 | | 0.23±0.16 | 0.64±0.53 |
| **p value (HFHZ vs HPUHZ)** | | |  | |
| Time | | | **<0.01** | |
| PUFA | | | 0.69 | |
| PUFA x Time | | | 0.95 | |
| **p value (HFHZ vs HFLZ)** | | |  | |
| Time | | | **<0.01** | |
| Zn | | | 0.16 | |
| Zn x Time | | | 0.39 | |

The values mean ± SD (n=9).
